# Fine-Tuning the Photocatalytic Activity of the Anatase {1 0 1} Facet through Dopant-Controlled Reduction of the Spontaneously Present Donor State Density

**DOI:** 10.1021/acsmaterialsau.4c00008

**Published:** 2024-03-26

**Authors:** Szymon Dudziak, Jakub Karczewski, Adam Ostrowski, Grzegorz Trykowski, Kostiantyn Nikiforow, Anna Zielińska-Jurek

**Affiliations:** † Department of Process Engineering and Chemical Technology, Gdansk University of Technology, G. Narutowicza 11/12, 80-273Gdansk, Poland; ‡ Institute of Materials Science and Nanotechnology, Gdansk University of Technology, G. Narutowicza 11/12, 80-273Gdansk, Poland; § Institute of Molecular Physics, Polish Academy of Science, Smoluchowskiego 17, 60-179 Poznan, Poland; ∥ Department of Chemistry of Materials, Adsorption and Catalysis, Faculty of Chemistry, Nicolaus Copernicus University, Gagarina 7, 87-100 Torun, Poland; ⊥ Institute of Physical Chemistry, Polish Academy of Sciences, Kasprzaka 44/52, 01-224Warsaw, Poland

**Keywords:** TiO_2_, crystal facets, doping, donor density, ROS

## Abstract

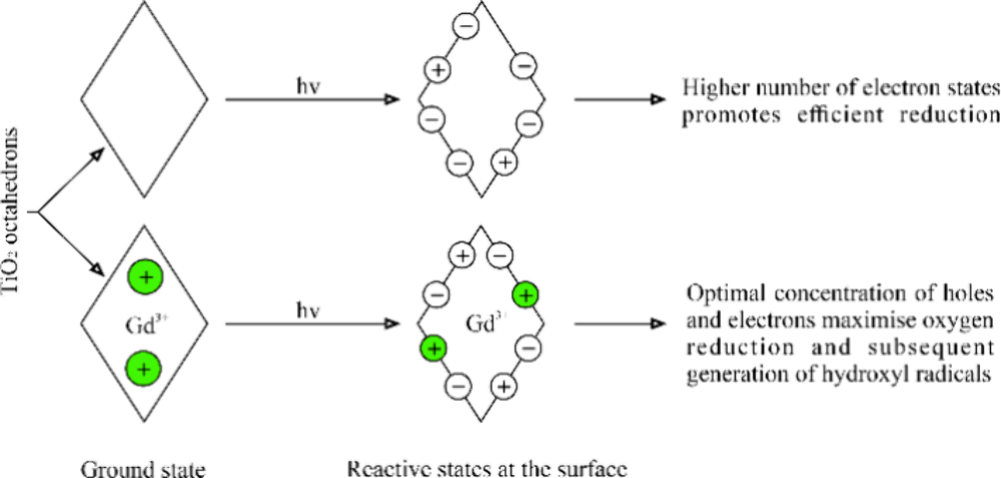

The present study highlights the importance of the net density of charge carriers at the ground state on photocatalytic activity of the faceted particles, which can be seen as a highly underexplored problem. To investigate it in detail, we have systematically doped {1 0 1} enclosed anatase nanoparticles with Gd^3+^ ions to manipulate the charge carrier concentration. Furthermore, control experiments using an analogical Nb^5+^ doped sample were performed to discuss photocatalytic activity in the increased range of free electrons. Overall results showed significant enhancement of phenol degradation rate and coumarin hydroxylation, together with an increase of the designed Gd/Ti ratio up to 0.5 at. %. Simultaneously, the mineralization efficiency, measured as a TOC reduction, was controlled between the samples. The observed activity enhancement is connected with the controlled decrease of the donor state density within the materials, being the net effect of the spontaneously present defects and introduced dopants, witch reduce hydroxylation and the hole trapping ability of the {1 0 1} facets. This allows to fine-tune multi-/single-electron processes occurring over the prepared samples, leading to clear activity maxima for 4-nitrophenol reduction, H_2_O_2_ generation, and ·OH formation observed for different donor densities. The optimized material exceeds the activity of the TiO_2_ P25 for phenol degradation by 52% (377% after surface normalization), showing its suitable design for water treatment. These results present a promising approach to boost photocatalyst activity as the combined result of the exposed crystal facet and dopant-optimized density of ground-state charge carriers.

## 1 Introduction

Photocatalytic degradation of emerging pollutants in water is one of the most important applications of semiconductor photocatalysis, with much attention being dedicated to finding new structures and their combinations to increase process efficiency. Within these studies, anatase TiO_2_ is often recognized as one of the most active photocatalysts and due to the high number of theoretical, experimental, and synthesis results, it became an important material to study photocatalytic reactions at their fundamental level. Recently, progress made in the preparation of anatase nanoparticles enabled the controlled synthesis of TiO_2_ photocatalysts exposing different crystal facets,^1−5^ which are used to study the effect of the photocatalyst’s surface on details of the photodegradation processes.^6−12^ It was found that atom arrangement at the photocatalyst interface plays a crucial role in the reactivity of the photogenerated charge carriers, with different redox potentials, charge carriers dynamics, preference to trap e^–^ or h^+^, and different trap densities being expected between different crystal facets.^13−18^ In this regard, the impact of the anatase surface structure on the efficiency of phenol degradation and mineralization was recently studied, which has shown that octahedral particles exposed with the {1 0 1} facets allow achieving improved mineralization efficiency than other nanostructures under UV–vis light irradiation.^19−21^ This fact was suggested to result from the increased reduction ability of the {1 0 1} facets^6,13,22^ that can stimulate O_2_ reduction to ·O_2_^–^ radicals, which are known to be effective ring-opening agents in AOP processes.^23^ However, this interpretation of the degradation ability of {1 0 1} facets makes it intriguing to what extent it depends on the simultaneous h^+^ transfer.

Moreover, in the case of photocatalyst design, the most common approach is to enhance charge carrier separation through the coexposition of multiple different crystal facets and possibly their selective doping or modification with the cocatalysts. The importance of crystal facets is constantly highlighted in different photocatalytic processes,^24−27^ and investigation of such facet-dopant interactions is an important direction of future works on photocatalyst design. In this regard, in the present study, octahedral anatase particles exposed with {1 0 1} facets were doped with 0.25–1.5% Gd as a possible electron-accepting dopant. The effect of the Gd/Ti ratio on phenol degradation efficiency and mineralization measured as a TOC reduction was investigated. The present study presents the importance of the net density of charge carriers at the ground state on the overall photocatalytic activity, which in photocatalysis can be seen as a highly underexplored problem.

## 2 Methods

### 2.1 Chemicals

Commercial P25 (Evonik, Germany), potassium hydroxide (POCH, ≥ 96%), 25% ammonia solution (POCH), ammonium chloride, Gd(NO_3_)_3_·6H_2_O, and NbCl_5_ (both from Sigma-Aldrich, ≥99%) were used during the preparation procedures. Phenol, *ortho-*hydroxyphenol, *para*-hydroxyphenol, benzoquinone, 4-nitrophenol, and 4-aminophenol were used for the photocatalytic tests as model pollutants and/or as standards for the calibration curves (all from Merck/Sigma-Aldrich with purity ≥98%). HPLC-grade acetonitrile and an 85% H_3_PO_4_ solution (both from Merck) were used for the mobile-phase preparation. Coumarin and 7-hydroxycoumarin (both from Sigma-Aldrich with purity ≥99%) were used as probe and calibration standards for the ·OH generation tests. All chemicals were used as received from the manufacturers.

### 2.2 Preparation of the Photocatalysts

Synthesis of the {1 0 1} exposed anatase octahedrons was performed using a two-step etching–rebuilding process, as reported before.^28,29^ In a typical procedure, 1 g of commercial P25 was treated with 40 cm^3^ of 8.5 M potassium hydroxide solution, using a 100 cm^3^ Teflon-lined stainless-steel autoclave placed in a laboratory oven, which was subsequently heated up to 200 °C. Total etching time, including approximately 1.5 h of heating, was set to be 16 h. Obtained products were centrifuged and then washed with water to a pH close to 7, followed by drying at 80 °C. Dried powders were hand-ground in an agate mortar, and 0.4 g of each one was introduced to the 200 cm^3^ reactor, together with 100 cm^3^ of NH_4_Cl/NH_4_OH buffer with pH set to 9 (0.3/0.3 M). The rebuilding step was further carried out for another 16 h at 210 °C. The final products were centrifuged, washed five times with water, and then dried at 80 °C.

For Gd/Nb doping, the calculated amount of Gd(NO_3_)_3_·6H_2_O/NbCl_5_ was introduced in the first step, together with P25. The designed Gd/Ti concentrations were as follows: 0.00, 0.25, 0.50, 0.75, 1.00, and 1.50 at. %. The Nb-doped samples were prepared with the designed concentration of Nb/Ti = 1.00 at. %.

### 2.3 Characterization of the Photocatalysts

The phase structure of the obtained photocatalysts was examined by using the powder XRD method with a Rigaku MiniFlex diffractometer. Analysis was performed using a Cu Kα source, within the 2θ range of 2–90° with the scanning step and speed of 0.05° and 1°·min^–1^, respectively. Rietveld refinement of the obtained anatase patterns was performed using X’Pert HighScore Plus software, including specimen displacement, background function, lattice constants, and profile parameters with anisotropic broadening. Morphology and elemental composition (Gd doping) were examined with the SEM observations under the FEI Quanta FEG 250 microscope equipped with the Apollo-X SSD spectrometer for the EDS analysis. The detection level for the analyzed elements, except for oxygen, was determined as 0.1 at. %. Before SEM/EDS analysis, a Au layer was deposited on the sample to help remove introduced excess electrons. Statistical analysis of the size distribution was performed based on at least 200 observations for each sample. Additional ICP-OES measurements were also performed concerning Ti and Gd presence in the prepared samples to obtain the total concentration of Gd with better accuracy (error estimated from three measurements). XPS analysis was performed using a PHI 5000 VersaProbe spectrometer with a monochromatic Al Kα radiation source. Deconvolution of the high-resolution spectra was performed with Casa XPS 2.3 software using a Shirley background and an asymmetrical Gaussian–Lorentzian function. The TEM images were recorded using a transmission electron microscope (Tecnai Osiris) equipped with a field emission gun (FEG), EHT = 200 keV, in TEM (Rio camera) and STEM (high-angle annular dark-field (HAADF) detector) modes. The TEM is equipped with an energy-dispersive X-ray spectrometer (EDS) with an energy resolution of 130 eV (Bruker Super-X). The preparation of samples proceeded in the following steps: sonication for 5 s of a few milligrams of sample in ethanol (99.8% anhydrous) using ultrasounds, applying a drop of the solution of 5 μL on a carbon-coated copper mesh with holes (Lacey type Cu 400 mesh, Plano), evaporating the solvent at room temperature, and then investigating the remaining dried powder stuck on the copper mesh/carbon layer. Electron paramagnetic resonance (EPR) spectra of powdered samples were recorded at 120 K by using a Bruker ELEXSYS spectrometer (X-band). The sample’s temperature was controlled and stabilized with a BVT 3000 Bruker Temperature Controller. To determine the paramagnetic ion concentration of the samples, the spectra were double integrated and compared with the intensity of a standard. Additional measurements were performed after *in situ* excitation of the sample with UV light for 60 min, and the recorded signal was compared to the ground-state measurement (collected directly before the excitation) in order to examine stable photogenerated species. The specific surface area of the obtained photocatalysts was determined using the BET isotherm method, measured with the N_2_ adsorption for the 10 points between the *p*/*p*_0_ range of 0.05 to 0.3. Measurements were performed at the temperature of liquid N_2_ by using a Micromeritics Gemini V analyzer. All samples were degassed at 140 °C for 3 h under N_2_ flow before the analysis. The UV–vis absorption spectra were analyzed using a Thermo Scientific Evolution 220 spectrometer with BaSO_4_ as the standard for the diffuse reflectance. The optical band-gap values of the samples were further calculated, based on Tauc’s method. Photoluminescence spectra were recorded using a Shimadzu RF-6000 spectrofluorometer, using a 300 nm excitation wavelength and a 400 nm cutoff filter at the emission side. Electrochemical analysis of the selected samples was performed using an Autolab PGSTAT204 potentiostat–galvanostat equipped with the FRA32 M module, 0.5 mol·dm^–3^ Na_2_SO_4_ solution as electrolyte, and screen-printed electrode systems (with working and counter electrodes made of carbon and reference electrode being Ag/AgCl). For electrode preparation, 5 mg of the prepared samples was ultrasonically dispersed in 0.5 cm^3^ of the 1:1 vol ethanol/water mixture, and subsequently, 15 μL of the suspension was drop-casted on the surface of the working electrode. The prepared layer was dried and then blocked with 5 μL of Nafion solution (1% in ethanol) and again dried for final measurements. The Mott–Schottky analysis of the samples was performed based on the electrochemical impedance spectroscopy data, collected using 10 mV amplitude of the AC signal and 1000 Hz frequency. The space charge capacitance (*C*) was calculated using the relation^30^*C* = −(2π × *f* × *Z*_im_)^−1^, where *f* is the frequency and *Z*_im_ is the imaginary part of the impedance. The density of the donor states (*N*_D_) was then calculated using the know relation with *C*:
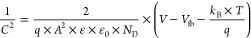
where *q* is the elementary charge, *A* is the interface area (simplified as the area of the working electrode), ε is the relative permittivity, ε_0_ is the vacuum permittivity, *V* is the applied potential, *V*_fb_ is the flat band potential of the electrode, *k*_B_ is the Boltzmann constant, and *T* is the temperature. To account for the possible errors during the electrode preparation, each sample was used to prepare two separate electrodes and the final values are presented as a mean with estimated standard deviation.

### 2.4 Photocatalytic Tests

The photocatalytic activity of the obtained samples was tested in the model reaction of phenol degradation. For a single run, 50 mg of the photocatalyst was introduced to 25 cm^3^ of 20 mg·dm^–3^ of phenol solution (prepared from the 500 mg·dm^–3^ stock solution) inside the quartz reactor. The reactor was then placed inside the black box, connected to the thermostat (20 °C), and magnetically stirred (600 rpm) under the 4 dm^3^·h^–1^ airflow for 30 min to achieve adsorption–desorption equilibrium. After this time, UV–vis light was introduced to the system using a 300 W xenon lamp, equipped with a water filter to cut off IR light. The reactor-lamp distance was set up before measurements to achieve a UVA (310–380 nm) flux intensity of 30 mW·cm^–2^ at the reactor border. A single process under light irradiation lasted for 30 min.

Additional tests of photocatalytic 4-nitrophenol reduction to 4-aminophenol were conducted to test the reactivity of the photogenerated electrons. The procedure was analogous to phenol degradation, except that 0.5 mM solution in methanol was used as the matrix and 25 mg of the photocatalyst was introduced instead of 50 mg. The solution was stabilized for 20 min under the Ar flow, instead of air, and was further irradiated without any gas flow present. Ability to generate ·OH radicals was tested during the 40 min process of coumarin degradation (0.5 mM), using 25 mg of the photocatalyst, under otherwise conditions identical to phenol degradation. Finally, generation of H_2_O_2_ was studied in the analogical conditions, with 25 mg of the photocatalyst, aeration increased to 10 dm^3^·h^–1^, and 5 cm^3^ of 2-propanol as a hole scavenger.

### 2.5 Analytical Procedures

All collected samples were passed through a 0.2 μm filter to remove photocatalyst particles and were further analyzed using a high-pressure liquid chromatography system. Both phenol and 4-nitrophenol analytic procedures were conducted using the C18 column, operating at 45 °C, and detection was based on external calibration using a diode-array detector. For the phenol measurements, a mobile phase consisting of (v/v) 0.700 acetonitrile, 0.295 water, and 0.05 H_3_PO_4_ solution (85% w/w) was used with the flow rate of 0.3 cm^3^·min^–1^. During the 4-nitrophenol measurements, the mobile phase was composed of (v/v) 0.700 water, 0.295 acetonitrile, and 0.05 of H_3_PO_4_ solution with a flow rate of 1 cm^3^·min^–1^.

After the phenol degradation tests, an additional sample was collected for the TOC analysis, using the Shimadzu TOC-L apparatus, and the residual concentration was compared to pure 20 mg·dm^–3^ phenol solution prepared without the addition of a photocatalyst.

Formation of 7-hydroxycoumarin was monitored by measuring the characteristic 454 nm emission intensity after excitation with 332 nm wavelength, using a Shimadzu RF-6000 spectrofluorometer after previous calibration with the prepared solutions. The detection limit was estimated approximately at 0.9 nmol·dm^–3^, based on the recorded signal being at least 3× higher than the average signal obtained for the blank sample. Simultaneous monitoring of the coumarin presence was performed based on the characteristic 278 nm absorbance maximum, as suggested in literature,^31^ after the previous dilution of the samples 2× with distilled water.

Determination of the H_2_O_2_ presence was monitored spectrophotometrically, using the iodometric method. Briefly, 1 cm^3^ of the filtered sample was mixed with 1 cm^3^ of KI solution (0.4 M) and 1 cm^3^ of potassium phthalate solution (0.2 M). After the mixture was left to react for 4 min in darkness, absorbance of the resulting solution was measured and H_2_O_2_ presence was determined from the 350 nm maximum based on the performed calibration.

### 2.6 Calculation of the Photonic Efficiency

Photonic efficiency of phenol degradation (ζ) was calculated for the 5 min process as the ratio between degraded phenol molecules and the amount of UVA (310–380 nm) photons introduced to the reactor, which can excite the TiO_2_ photocatalyst:
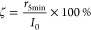
where *r*_5 min_ is the rate of phenol disappearance at 5 min of the process (μmol·min^–1^) and *I*_0_ is the incident UVA photon flux that reaches the reactor border (μmol·min^–1^). *I*_0_ is estimated to be 24.937 μmol·min^–1^, based on the controlled UVA flux intensity, as well as lamp emission spectrum and flux distribution at the reactor border, which for the used setup is reported in detail elsewhere.^32^ For all experiments, the same lamp-reactor configuration was used.

### 2.7 Computational Details

The effect of Gd doping on the electronic structure of bulk TiO_2_ anatase, as well as {1 0 1} surface slab model, was investigated computationally, using the Quantum Espresso software package.^33,34^ The 2 × 2 × 2 anatase supercell was used for the bulk calculations, while for the slab model, the exposed area was set to consist of eight Ti atoms, which were further repeated forming four consecutive layers, separated by 20 Å of vacuum (the model is presented in Figure 4). In this manner, both models have the same number of 32 Ti atoms. For the Gd doping, a single Ti atom at the center of the model was replaced with Gd, which should correspond to the Gd/Ti concentration approximately 3 at. %. Lower Gd concentration was not investigated due to the computational cost. Calculations were performed using the projector augmented wave (PAW) method with Perdew–Burke-Ernzerhof (PBE^35^) functionals, and the Hubbard parameter *U* was added to account for the on-site Coulomb interactions to obtain reasonable band gap values (*U* = 6.8 eV for Ti/Gd and 3 eV for O were selected, based on the values reported before for TiO_2_ and ZnO systems doped with Gd^36−39^). The Monkhorst–Pack *k*-point mesh of 4 × 4 × 3 and 5 × 5 × 1 was used for the bulk and surface slab calculations, respectively. The waveplane was expanded to 700 eV for the pure TiO_2_ models, and to the 1100 eV for the Gd-modified system. All models were fully relaxed, including all atoms, using the Broyden–Fletcher–Goldfarb–Shanno (BFGS) method to a convergence threshold of 10^–3^ Ry·Bohr^–1^. During the bulk calculations, cell dimensions were allowed to optimize and were further fixed for the slab model. Geometric optimization was followed with the density of states (DOS) determination under the same *k*-point mesh.

## 3 Results and Discussion

### 3.1 Photocatalyst Structure and Phenol Degradation

**Figure 1:**
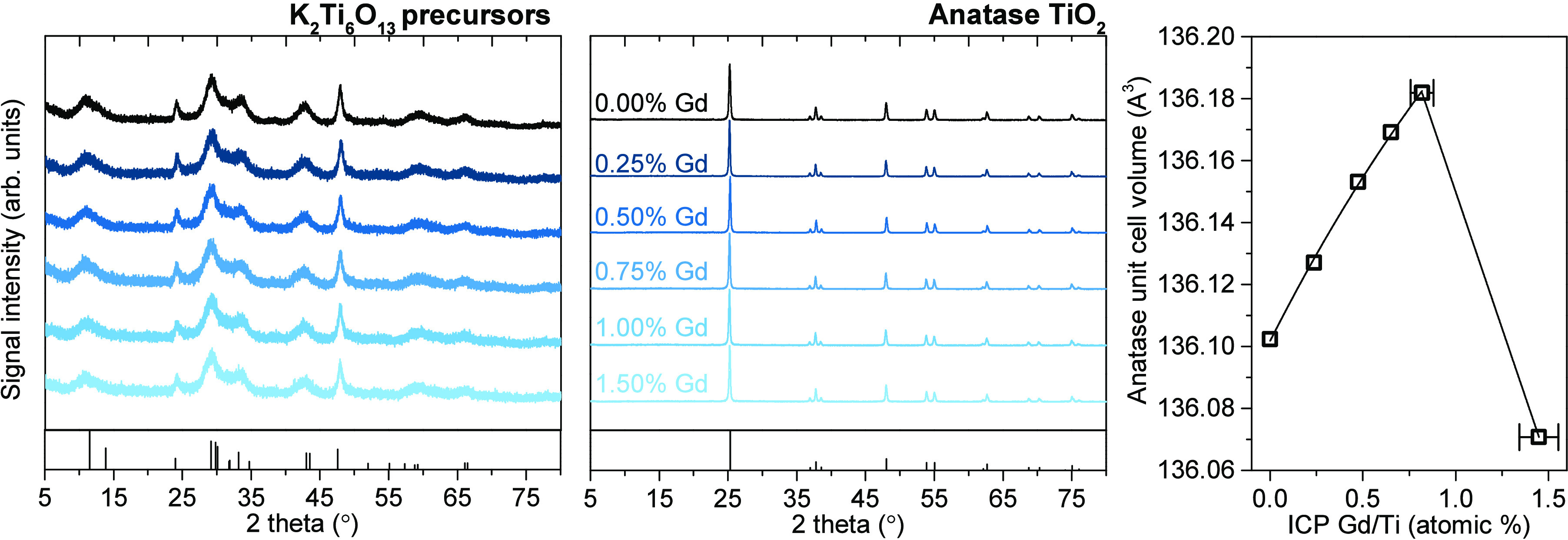
XRD patterns of the K_2_Ti_6_O_13_ precursors and resulting Gd-modified anatase photocatalysts with corresponding changes in the anatase unit cell volume, obtained from the Rietveld-refined patterns. Detailed results of the refinement are shown in the ESI (Figure S2). Standard peak patterns of both phases are marked in the boxes at the bottom.

The formation of the designed nanoparticles was based on the two-step transformation of commercial TiO_2_ (P25) to its K_2_Ti_6_O_13_ titanate and subsequent rebuilding of the TiO_2_ structure in the controlled environment.^28,29,40^ During this process, Gd should incorporate and diffuse into the transforming TiO_2_ lattice in a manner analogical to the one observed before for Ni.^41^ This is also reasoned by the calculations of Mulwa et al.,^37^ as well as Chen et al.,^36^ who have shown that Gd substitution in place of Ti should be energetically possible inside the anatase structure. As shown in Figure 1, XRD analysis confirmed the formation of both K_2_Ti_6_O_13_ and TiO_2_ phases and the final product was found to be pure anatase. No presence of the secondary Gd phase was observed in the XRD patterns of both precursors and final products. Furthermore, Gd presence was confirmed with the ICP measurements, as summarized in Table 1, with the overall Gd concentrations being close to the nominal ones. Following this, Rietveld refining of the patterns revealed a systematic increase of the anatase unit cell together with the ICP-determined Gd presence up to approximately 1.00% of the Gd/Ti concentration. This is in accordance with the Gd size when substituting Ti. However, it might be noted that for the 1.50% sample, the trend is clearly broken, which might indicate that Gd incorporation became limited at this concentration.

**Table 1:** Determined Gd Presence and BET Surface Area of the Photocatalysts and Their Activity toward Phenol Removal during the 30 min Process

sample	ICP Gd/Ti (at. %)	EDS Gd/Ti (at. %)	XPS Gd/Ti (at. %)	BET (m^2^·g^–1^)	observed *k* (min^–1^)	normalized *k* (min^–1^·m^–2^)^a^	ζ at 5 min (%)	TOC removal (mg·h^–1^)	norm. TOC rem. (mg·m^–2^·h^–1^)^a^
0.00%^b^	0.00	0.0	0.00	18.1	0.130	0.144 ± 0.014	1.52	0.574	0.667 ± 0.093
0.25%	0.236 ± 0.018	0.2 ± 0.2	1.75	15.6	0.145	0.186 ± 0.019	1.98	0.530	0.678 ± 0.095
0.50%	0.475 ± 0.036	0.4 ± 0.2	4.76	15.9	0.163	0.204 ± 0.020	2.19	0.530	0.667 ± 0.095
0.75%^b^	0.652 ± 0.051	0.5 ± 0.2	4.43	14.8	0.112	0.151 ± 0.015	1.53	0.447	0.603 ± 0.084
1.00%	0.819 ± 0.062	0.8 ± 0.2	6.68	19.8	0.134	0.136 ± 0.014	1.63	0.577	0.582 ± 0.082
1.50%	1.450 ± 0.105	1.0 ± 0.2	8.91	16.8	0.100	0.119 ± 0.012	1.51	0.308	0.368 ± 0.052
P25^b^	N/A	N/A	N/A	52.7	0.107	0.043 ± 0.004	1.28	0.623	0.237 ± 0.033

a Relative error estimated at 10% for normalized *k* and 14% for normalized TOC removal. Details in the SI (Section 3, Figure S3 and Table S1).

b Mean values from two measurements, performed for error estimation (except ICP and XPS).

**Figure 2:**
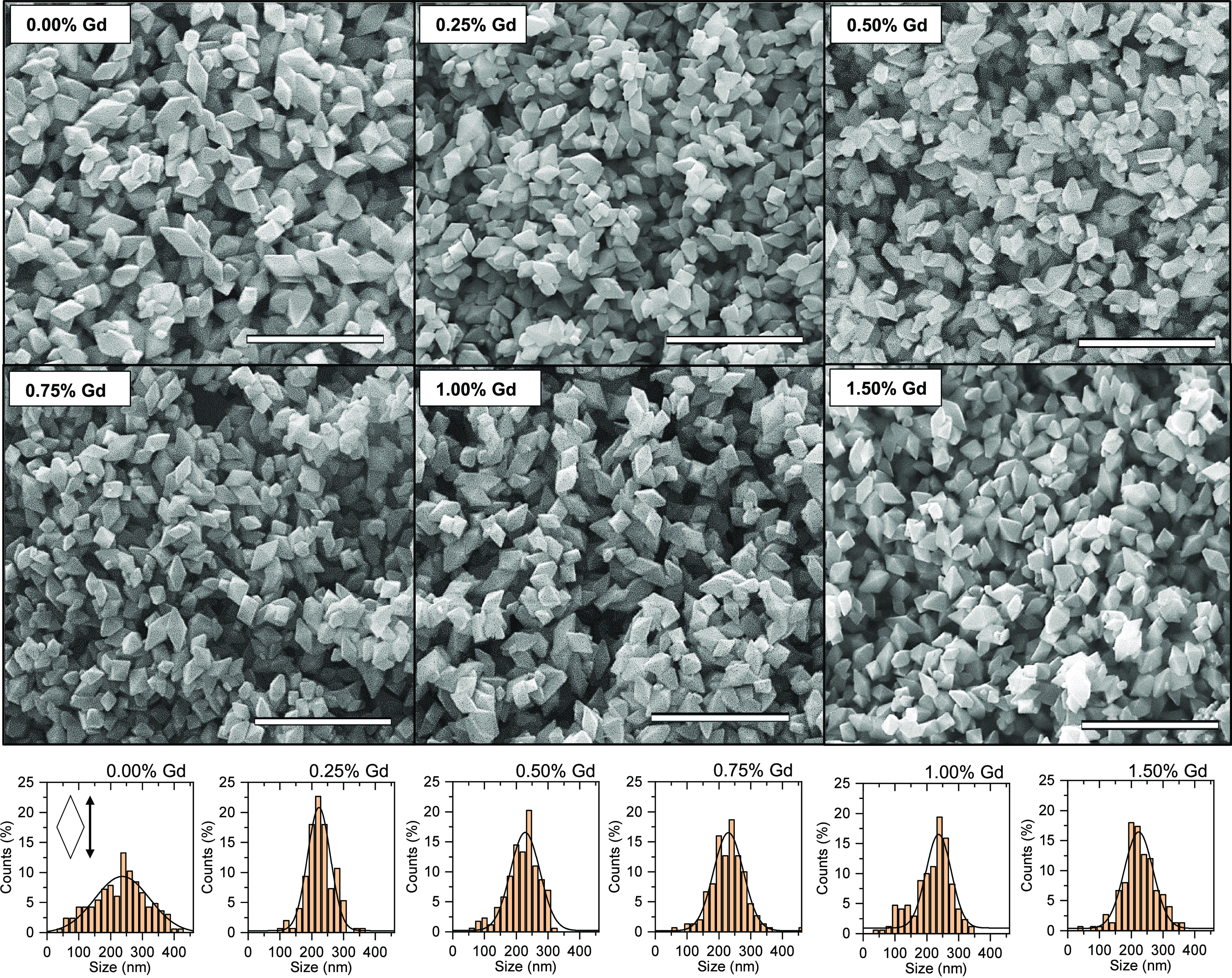
SEM images and corresponding size distributions of the {1 0 1} exposed anatase nanoparticles with different Gd/Ti concentration. Scale bars are 1 μm.

**Figure 3:**
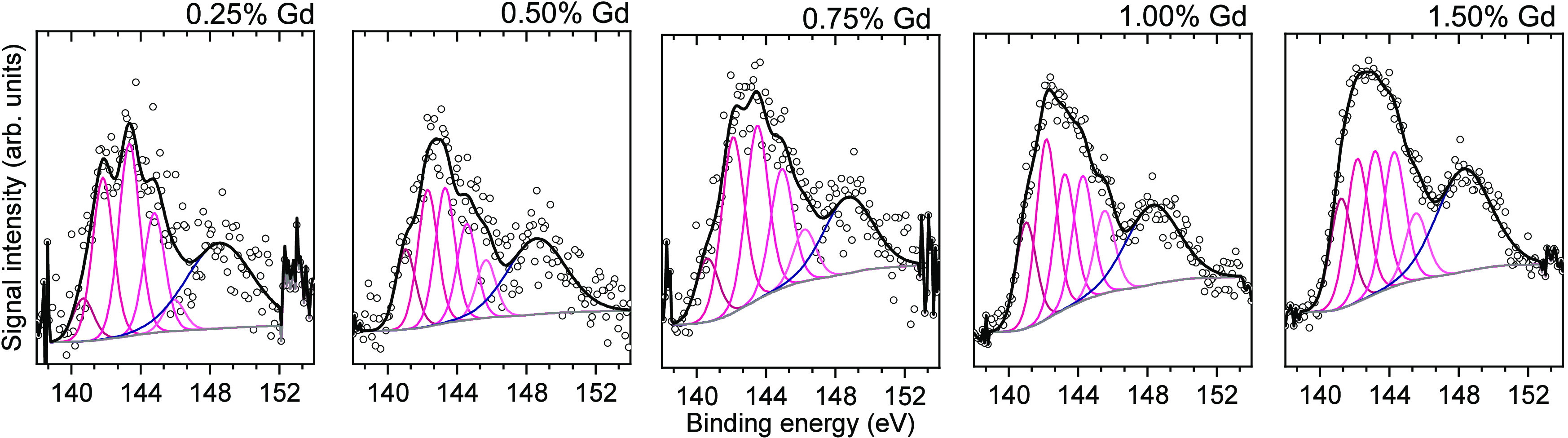
Deconvolution of the XPS signals corresponding to the Gd 4d states, with multiplet splitting of the 4d_5/2_ states presented with magenta lines for each sample.

Following the XRD measurements, SEM images have shown the formation of octahedral nanoparticles for all of the analyzed samples, which is in agreement with the expected exposure of the {1 0 1} facets. As shown in Figure 2, differences between the samples are limited and the orientation between exposed facets matches the theoretical one between the different {1 0 1} planes (Figure S1). Growth of the designed nanostructures was directly controlled by the environment of NH_4_Cl/NH_4_OH, which prevents pH change due to the K release from dissociating K_2_Ti_6_O_13_, therefore providing stable growth conditions. No clear differences in the morphology are observed between samples, with only slight differences observed in the determined size distributions, as presented in Figure 2. Specifically, for samples with the 0.00 and 1.00% of Gd, a larger fraction of particles <100 nm is observed, which might influence on the available surface area. Therefore, to further consider the possible effect of the exact surface development on the photocatalytic activity,^42^ the BET method was applied and the results of the analyses are presented in Table 1. Overall, the BET results matched determined size distributions well, with slightly larger areas determined for both the 0.00 and 1.00% samples. Following the SEM observations, additional EDS analysis confirmed the presence of Gd for all modified samples without additional signals. However, a small deviation between the EDS and ICP concentrations was noticed, especially for the most-modified sample. Therefore, both these analyses were completed with the XPS measurements, which has indicated significant enrichment in the surface Gd concentration, as summarized in Table 1. This was followed by determination of the position for the Gd 4d and Gd 3d signals. As presented in Table S2, the peak position of both these states is visibly shifted to the higher binding energies, especially in the case of the 4d states (142.7–143.0 eV for the prepared samples, compared to the 142–142.5 eV reported for Gd_2_O_3_,^43−45^ 141.7 eV reported for Gd(OH)_3_,^46^ and 140–141 eV reported for metallic Gd^44,45^). Moreover, detailed deconvolution of the Gd 3d states has shown multiplet splitting of the 4d_5/2_ peak with five expected states (magenta lines in Figure 3); however, their relative intensities are unusual, with high-energy bands being greatly suppressed, compared to the high-quality Gd compounds.^47,48^ Although detailed analysis of these multiplet states is beyond the scope of this work, it is known that they originate from the interactions between created photohole and 4f electrons and the alternation of the resulting peak shape strongly suggests that the local environment of the Gd ions might be different in the case of the prepared samples. No other significant differences between the samples were observed during XPS analysis, as presented in Figure S4.

Ultimately, both observations about Gd 4d/5d peak position and shape connect well with the expected role of Gd^3+^ as a substituent within the anatase lattice. However, significant accumulation of Gd at the surface simultaneously indicates that its diffusion deeper into the bulk structure is difficult under accepted preparation conditions. Therefore, in order to further investigate formation of a possible surface species and differences in the Gd distribution, samples 0.00, 0.50, and 1.50% were investigated in detail under TEM. As presented in 4a,b, these observations revealed prepared octahedrons to be single-crystalline structures with a terminal structure matching the expected exposition of the {1 0 1} facets. Furthermore, no difference between the overall was noticed, indicating that no Gd species at the surface could be clearly observed on the octahedron's surface (Figure S5).

**Figure 4:**
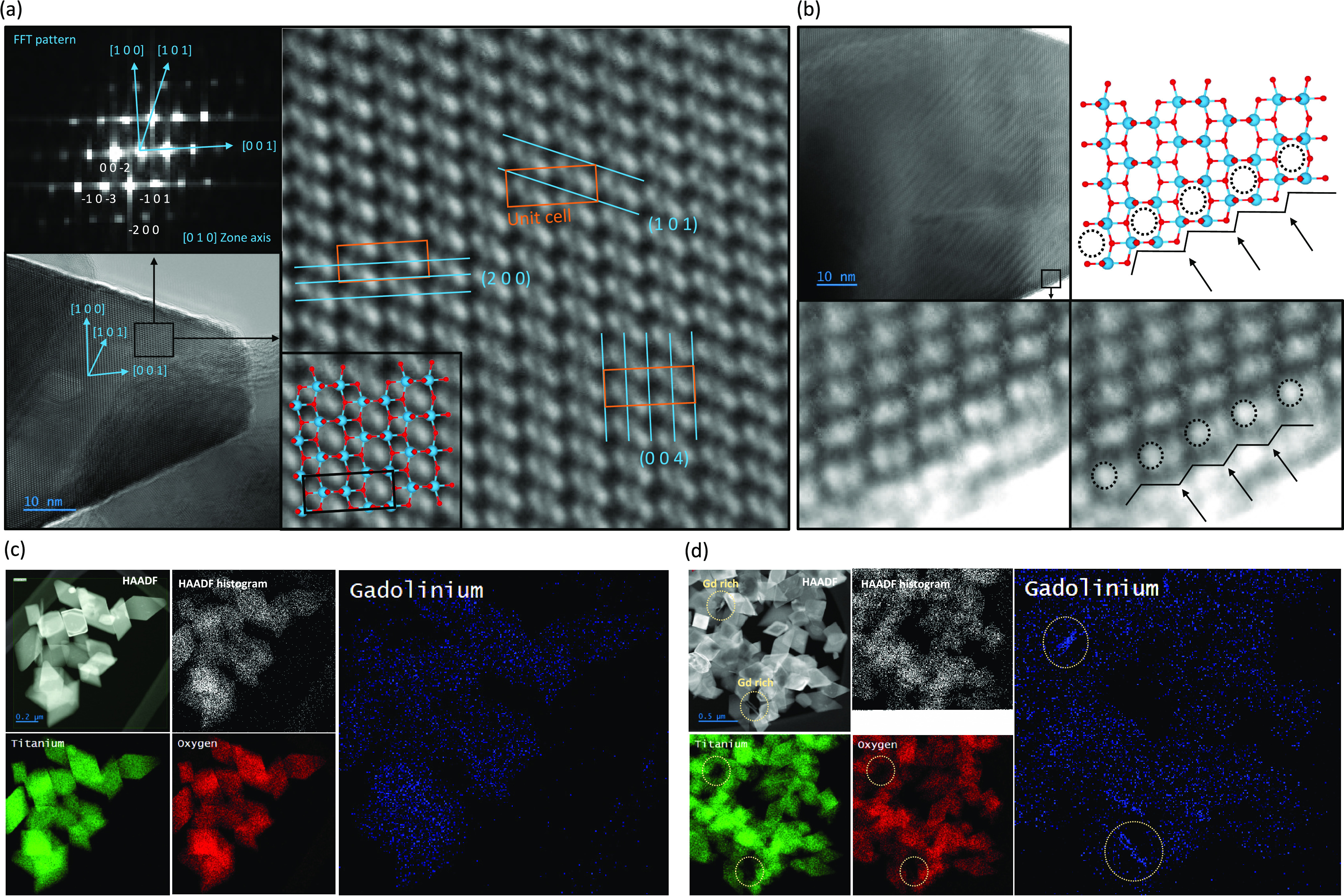
HR-TEM image of the selected TiO_2_ octahedron (a) with the corresponding FFT diffraction pattern and a detailed analysis of the crystal structure orientation. Closeup of the crystal structure observed at the interface, together with the scheme of the perfect, bulk-cut {1 0 1} termination (b). EDS and HAADF mapping of the elements observed for the 0.50% (c) and 1.50% (d) modified samples.

However, as presented in Figure 4d, in the case of the 1.50% modified sample, EDS mapping revealed Gd accumulation into independent structures outside the anatase crystallites. This has led to the nonuniform Gd distribution, with regions of bare TiO_2_ occasionally observed in the proximity of these Gd-rich particles; however, modified Gd octahedrons are still present within the sample. No such Gd-rich structures were observed in the case of the 0.50% sample, as presented in Figure 4c. These results indicate that for the increased Gd concentration, precipitation of the independent structure became more favored that distributed within the anatase lattice. Taking into account clear surface enrichment of Gd, this is in accordance with a difficult Gd diffusion into the anatase structure, which increases the probability of Gd_2_O_3_/Gd(OH)_3_ nucleation after the critical concentration of Gd^3+^ ions was locally exceeded. This also explains the nonlinear change in the anatase unit cell observed for the 1.50% doped sample, simultaneously suggesting that for other samples, such precipitation of the Gd structures should be limited. Ultimately, concerning morphology, crystal structure, and elemental composition, increasing Gd amount is the only consistent change between the samples up to 1.00% of the designed Gd/Ti concentration, with a clear Gd gradient between the surface and bulk structures being noted in all cases and additional precipitation of the other phase (below XRD sensitivity) happening only for the 1.50% sample. Overall, samples were concluded as suitable for discussing the role of Gd as a dopant of the {1 0 1} exposing TiO_2_ nanostructures.

**Figure 5:**
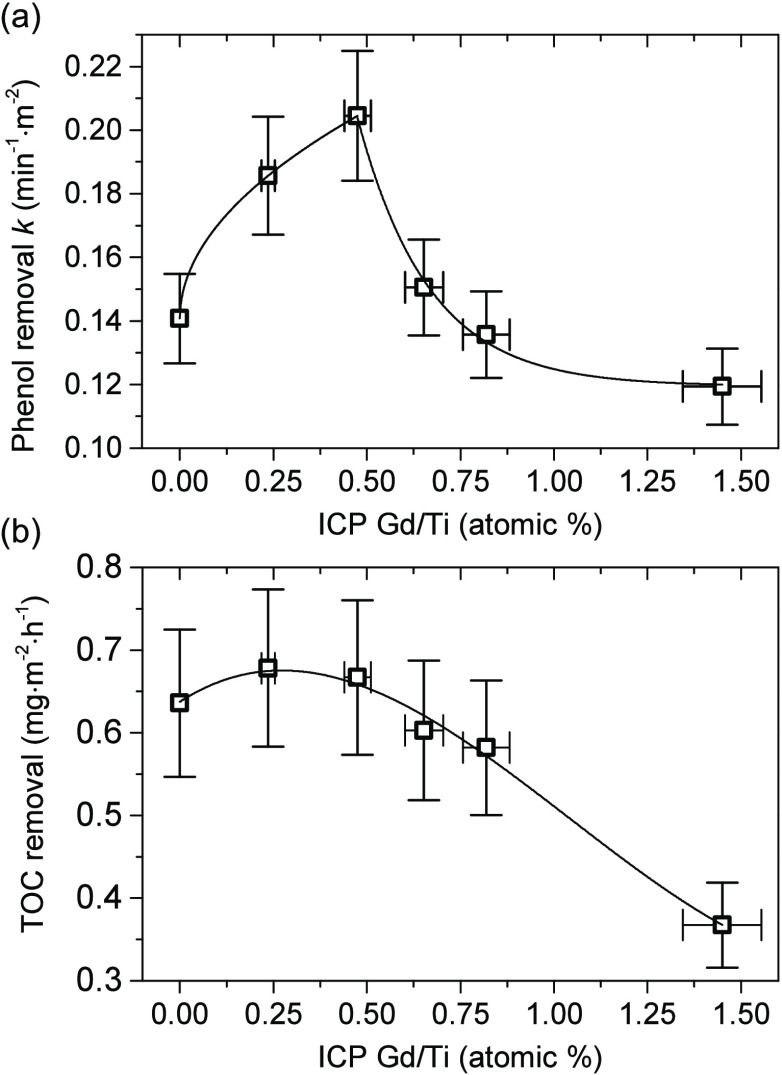
Surface-normalized results of photocatalytic phenol degradation for the prepared Gd-modified samples: (a) rate constant assuming I order kinetics and (b) TOC removal after 30 min of irradiation.

Following initial characterization, prepared samples were tested for phenol degradation, as a model aromatic pollutant in the aqueous phase. Figure 5 shows surface-normalized results of this process during the 30 min process under continuous UV–vis irradiation using a Xe lamp with a water filter as the light source. All of the obtained photocatalysts exhibit very good performance, which is visibly higher than that of the well-known TiO_2_ P25, as shown in Table 1, including rate constants *k* and calculated photonic efficiencies ζ. Furthermore, for samples with the designed Gd concentrations of 0.25 and 0.50%, an increase of the photoactivity is observed while simultaneous removal of the organic carbon remained at a similar level when compared to the unmodified {1 0 1} sample. The highest overall activity was observed for the sample with the designed 0.5% of Gd, which allowed it to degrade over 50% of phenol only after 5 min of the process (Figure S6). For higher amounts of Gd, a decrease in both degradation and mineralization efficiency is observed, which shows that optimal Gd concentration lies somewhere between 0.25 and 0.75% of the designed Gd/Ti ratio. Noteworthily, a significant decrease of the TOC removal for the sample with a nominal 1.50% concentration of Gd might also be connected with the observed precipitation of the Gd-rich structures along TiO_2_. Detailed results of the photocatalytic activity are presented in Figures S6 and S7a. Finally, sample 0.50% was additionally tested for the stability of the photocatalytic performance. These tests have indicated limited changes in the activity due to the sample storage and cycling but have indicated a significant effect of surface purification between each process. As shown in detail in Figure S7b, repeated experiments after approximately a year of storage have shown slightly reduced activity, overall within the expected error range (cycle I). Starting from this point, simple rinsing of the sample before introduction to the new phenol solution resulted in a significant decrease of the activity (cycle II), which was further returned near the original level after multiple rinses with deionized water and reactor cleaning (cycle III). After similar pretreatment, the activity was unchanged during cycle IV.

Overall, these results suggest stable operation of the prepared structures but highlight their high sensitivity to the possible residual contaminants left after the previous process.

### 3.2 Band Structure Analysis

**Figure 6:**
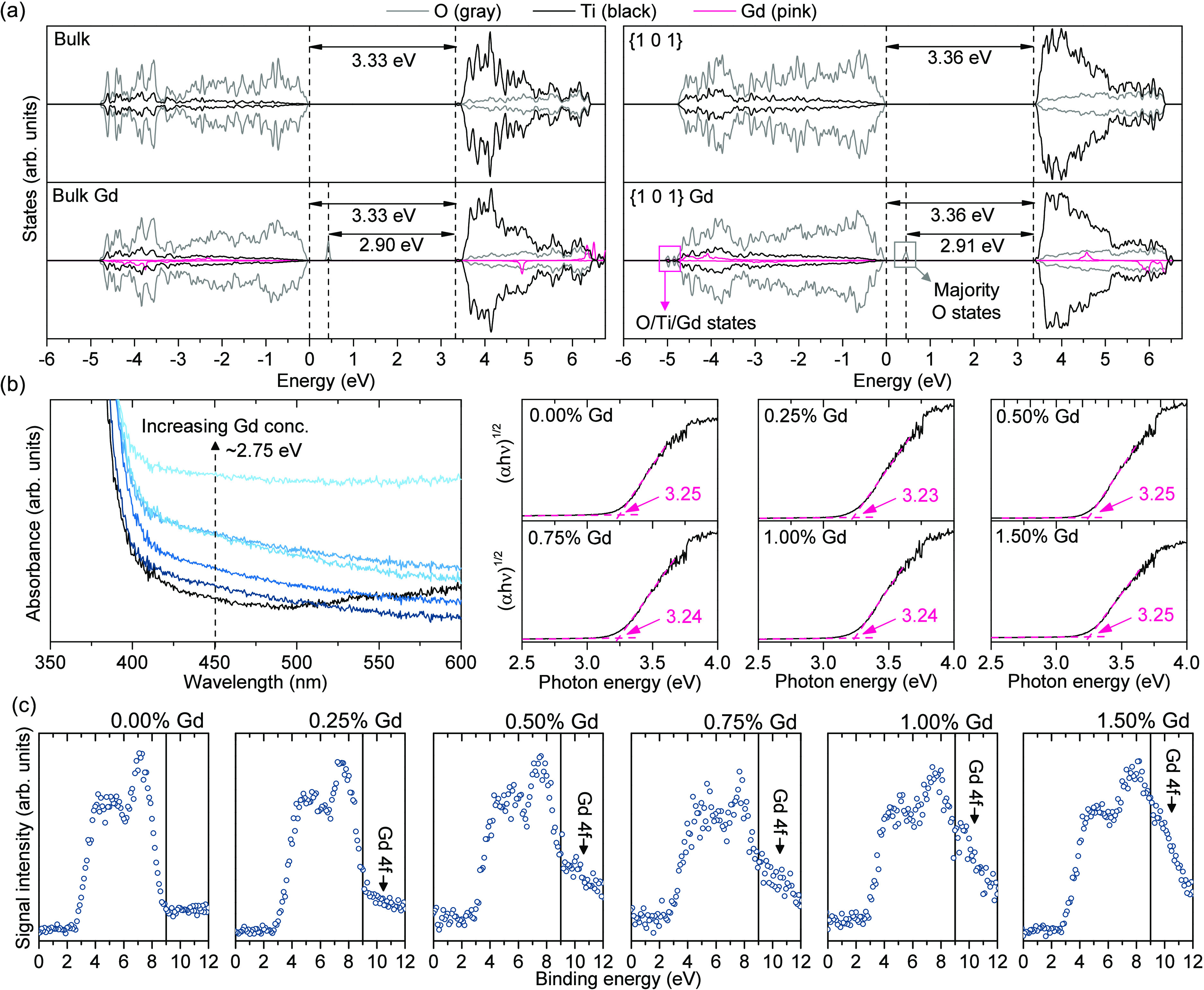
Simulated density of states distribution for pure and modified models of anatase bulk structure and {1 0 1} surface (a), together with the absorbance results in the proximity of a main valence-to-conduction band transition in the obtained TiO_2_ photocatalysts and the exact values of the main band gap, determined using Tauc’s method (b). Valence band scans of the prepared samples were performed during the XPS analysis (c).

Since initial characterization has not indicated formation of any separate Gd phase (except for the 1.50% sample), the increase of the photocatalytic activity observed for the lower Gd concentrations is attributed to the doping effect of Gd^3+^ into the anatase crystal structure. To investigate this explanation further, DOS calculations were performed for the Gd-doped bulk and {1 0 1} surface slab models of anatase. As shown in Figure 6a, in both cases, the presence of Gd resulted in the appearance of additional bands slightly above the valence band (VB) edge, which comprise most of the O 2p states. Simultaneously, no change in the valence-to-conduction gap is observed and the Gd states resonate with the low-energy VB states and high-energy conduction band states, respectively. Such results are in general agreement with the previous simulations of Gd-doped anatase.^36,37,49^ However, since no significant difference is observed between bulk and surface models, it also shows that no surface-specific states should be formed simply as the results of Gd introduction to the {1 0 1} exposing octahedrons.

Performed DOS simulations were followed by absorbance studies to experimentally confirm predicted band structures. As shown in Figure S8, obtained spectra are very consistent between the samples, which is in agreement with a very low concentration of introduced Gd. Nevertheless, detailed investigation of the spectra showed that Gd presence results in the relative increase of the absorbance between 410 and 500 nm (approximately 3–2.5 eV), which is consistent with the predicted formation of additional states above the VB edge. Simultaneously, for the unmodified TiO_2_ structure, a slight increase of the absorbance is observed up to 600 nm, which could be attributed to the presence of the excess electrons inside its structure.^6,50^ Since anatase TiO_2_ is an n-type semiconductor, the presence of some excess e^–^ is reasonable for the unmodified sample. Noteworthily, for sample 1.50%, a uniform increase of the absorption in the full investigated range might result both from the continuous distribution of the defect states formed for higher Gd concentration and from precipitation of additional Gd-rich particles with different optical properties. Outside these features, the main valence-to-conduction band transition is not affected by the Gd presence, as observed based on Tauc’s method.^51,52^ The summation of the absorbance analyses is shown in Figure 6b. Finally, DOS simulations have also predicted the appearance of additional states due to the Gd presence below the VB end, in the region also typical for other Gd-bearing compounds. Contrary to the states above the VB edge, these show significant contribution of the Gd 4f electrons, hybridized with the O 2p and Ti 3d states. Interestingly, in this case, the effect is not predicted for the bulk structure. Although due to their high binding energy it is unlikely that these states would affect photocatalytic performance of the samples, their presence was clearly observed during the XPS analysis, as presented in Figure 6c, additionally confirming the expected band structure of the sample.

### 3.3 Ground-State Defects and Density of Donor States

**Figure 7:**
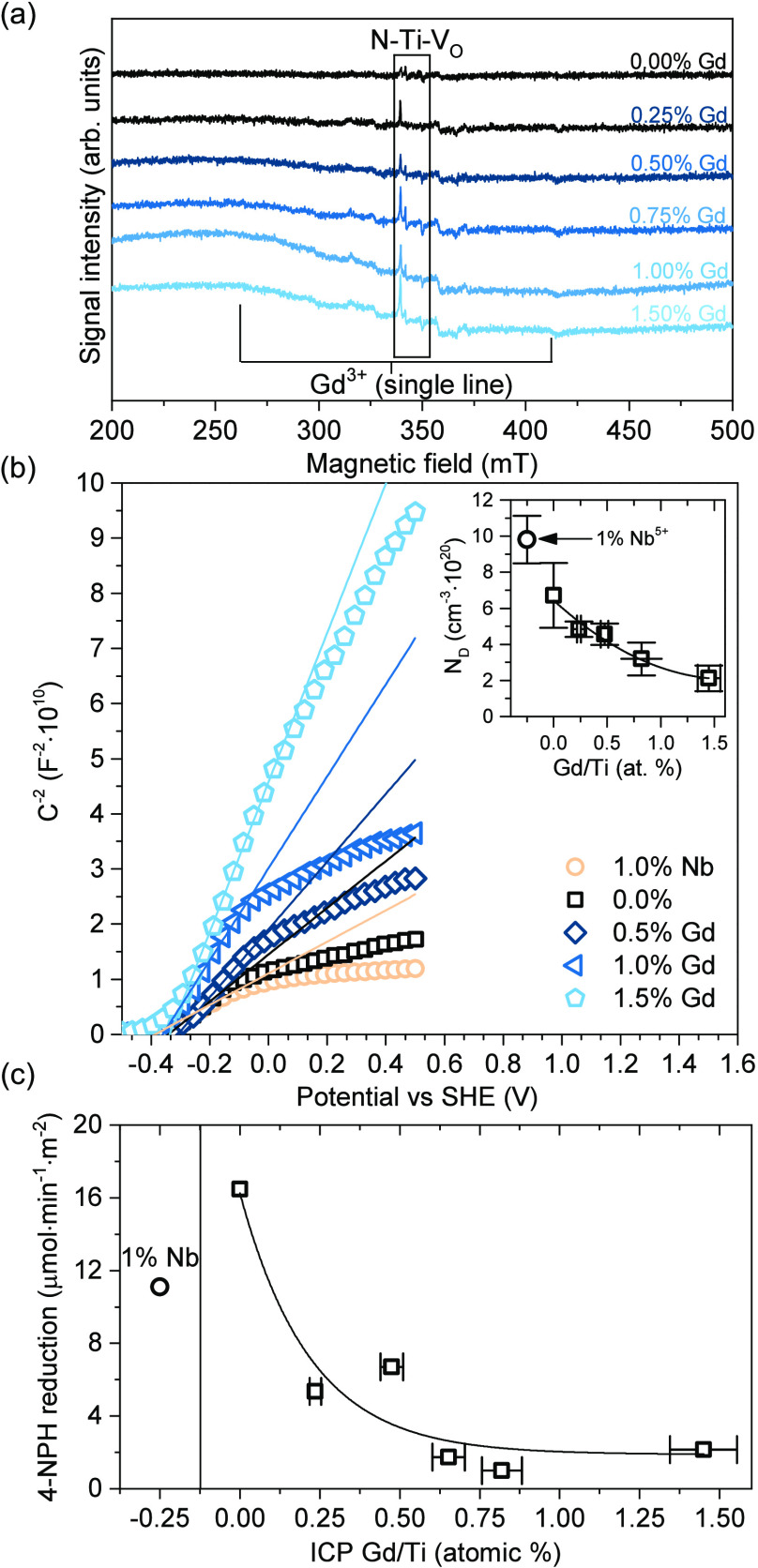
X-band EPR spectra of the prepared materials measured at 120 K (a). Mott–Schottky plot of the selected samples (mean from the two electrodes; details shown in the SI. The curve for the 0.25% sample was not shown here for clarity) and corresponding density of donor states *N*_D_ (b). Surface-normalized results of photocatalytic 4-nitrophenol (4-NPH) reduction over prepared samples (c).

Combined DOS and absorbance studies confirmed that Gd creates additional energy states above the VB top. Although the exact position of these states could be questioned (e.g., some studies predict overlapping of such states with the VB and thus no formation of new bands^36^), the fact that Gd affects DOS distribution around the VB edge is consistent for our results and other reports.^36,37,49^ Such an effect is characteristic of the electron-accepting dopants, which agrees with the Gd^3+^ valency when substituting Ti^4+^, and it remains the same for the bulk and the {1 0 1} surface structure of anatase. Furthermore, these results were followed by the detailed analysis of the possible defects within the prepared materials, based on the low-temperature EPR measurements. As presented in Figure 7a, the X-band spectra showed multiple features assigned roughly to the three defect types. First, the Gd-modified samples showed the appearance of a single, asymmetric broad line with an effective *g*-factor of *g* ≈ 2.07. An increase in the integral intensity of this line is observed as the concentration of gadolinium in the sample rises, indicating the incorporation of Gd^3+^ ions at Ti^4+^ sites within the TiO_2_ lattice.^53,54^ Second, all samples showed the appearance of additional signals with complex resonance spectra approximately between *g* ≈ 2.5 and *g* ≈ 1.6. These signals appeared systematically in the samples, without a clear correlation between their intensity and Gd presence. Noteworthily, analogical observations were made during the XPS analysis, where no correlation between Gd presence and, e.g., V_O_ signals, was noticed. This suggests that these defects originate due to the accepted preparation procedure and/or utilized substrates, leading to their relatively random distribution between the samples. Based on the existing studies, these could be roughly ascribed to the possible combination of vanadium impurities^55,56^ and N-including defects (signals with *g* = 2.00, 1.99, and 1.94, as reported previously,^57,58^ in the region highlighted with a rectangle); however, due to the low concentration, their strict identification is not obvious at the moment.

Ultimately, although qualitatively similar, EPR analysis revealed a quite complex, low-concentration defect structure of the prepared materials, with multiple species possibly influencing the final properties. Due to the possible interactions between these species, including mutual compensation of the associated charges, the quantitative effect of the introduced Gd^3+^ on the concentration of free carriers might not be obvious. In this regard, conductivity-type and detailed analysis of the free carrier concentration was performed based on the Mott–Schottky analysis of the 0.00, 0.25, 0.50, 1.00, and 1.50% Gd samples. Furthermore, to investigate this problem deeper, additional measurements of the analogical material doped with 1% of Nb^5+^ were also performed, in order to increase the number of free electrons compared to the 0.00% sample. We note that detailed preparation and characterization of the {1 0 1} exposing samples doped with different Nb^5+^ concentrations was recently reported,^32^ and overall electron-donating character of Nb is well documented when introduced to the TiO_2_ materials.^50,59,60^ Therefore, here we use it only as a control to prepare the Gd-doped series, with basic characterization of this sample presented in the SI (Figure S9). As shown in Figure 7b, all materials show an n-type character, confirmed with the positive slope of the fitted lines, resulting from the presence of donor states within the material (*N*_D_). Furthermore, the increase of the Gd amount clearly increases the slope of the fitted functions in the *C*^2–^(*E*) graph, which results from the decrease of *N*_D_. Although it is noteworthy that the calculated donor concentrations are likely overestimated, e.g., due to the actual contact area being larger than the area of the deposited layer, the trend of *N*_D_ reduction together with the Gd amount is clearly observed, as shown in the inset of Figure 7b. Since all materials have the same particle morphology and similar specific surface areas, this effect can be reasonably attributed to the Gd presence. Finally, this is further justified by the estimation of the possible error, based on the measurements of two separate electrodes, which further prove that the Gd effect is significant for this observation, independently of the possible contribution of other defects (detailed results are shown in Figure S10). Finally, control measurements for the Nb-doped samples confirmed the opposite effect, clearly increasing *N*_D_, compared to the unmodified material.

Reduction of the donor state density is strictly connected with the decrease in the number of free electrons within the material, which would imply that the reduction ability of these materials might be hindered compared with the unmodified sample. Indeed, this could be observed when testing the samples in the reaction of 4-nitrophenol (4-NPH) reduction to 4-aminophenol in methanol. Nitrophenol-to-aminophenol reduction is known to be a six-electron process in total;^61,62^ therefore, even a slight decrease in the number of reacting e^–^ is expected to influence the reaction efficiency. As shown in Figure 7c, for all Gd-modified samples, a significant decrease in the reaction yield is observed, with the relative decrease ranging from approximately 64% up to 95% of inhibition, when compared to the unmodified {1 0 1} sample. Since the observed effect is both significant and systematic for the modified samples, these results were left without error estimation. Noteworthily, a similar effect can be suggested to result from the hypothetical electron transfer from the anatase phase to the ultrafine Gd-species deposited on the surface, assuming that the Gd phase would be less reductive than TiO_2_. However, in such a case, a significant decrease in the observed recombination should be observed, which is not supported by photoluminescence measurements. As shown in Figure S11, after excitation with the 300 nm wavelength, emission spectra of all samples are very similar and show no clear correlation with the Gd presence, observed *N*_D_ value, or photocatalytic activity. In this regard, possible charge separation as the main mechanism of the observed results is not supported. Ultimately, overall results agree that introduced Gd acts as an electron-accepting dopant inside the prepared photocatalysts, reducing the number of spontaneously present donor states and reacting excess electrons.

Noteworthily, the Nb^5+^ presence also hindered 4-nitrophenol reduction, in accordance with the previous reports; however, the effect is less significant than in the case of Gd^3+^.

### 3.4 Discussion on the Photocatalytic Activity—Light-Induced ·OH/H_2_O_2_ Generation and EPR Monitoring of Hole Trapping

**Figure 8:**
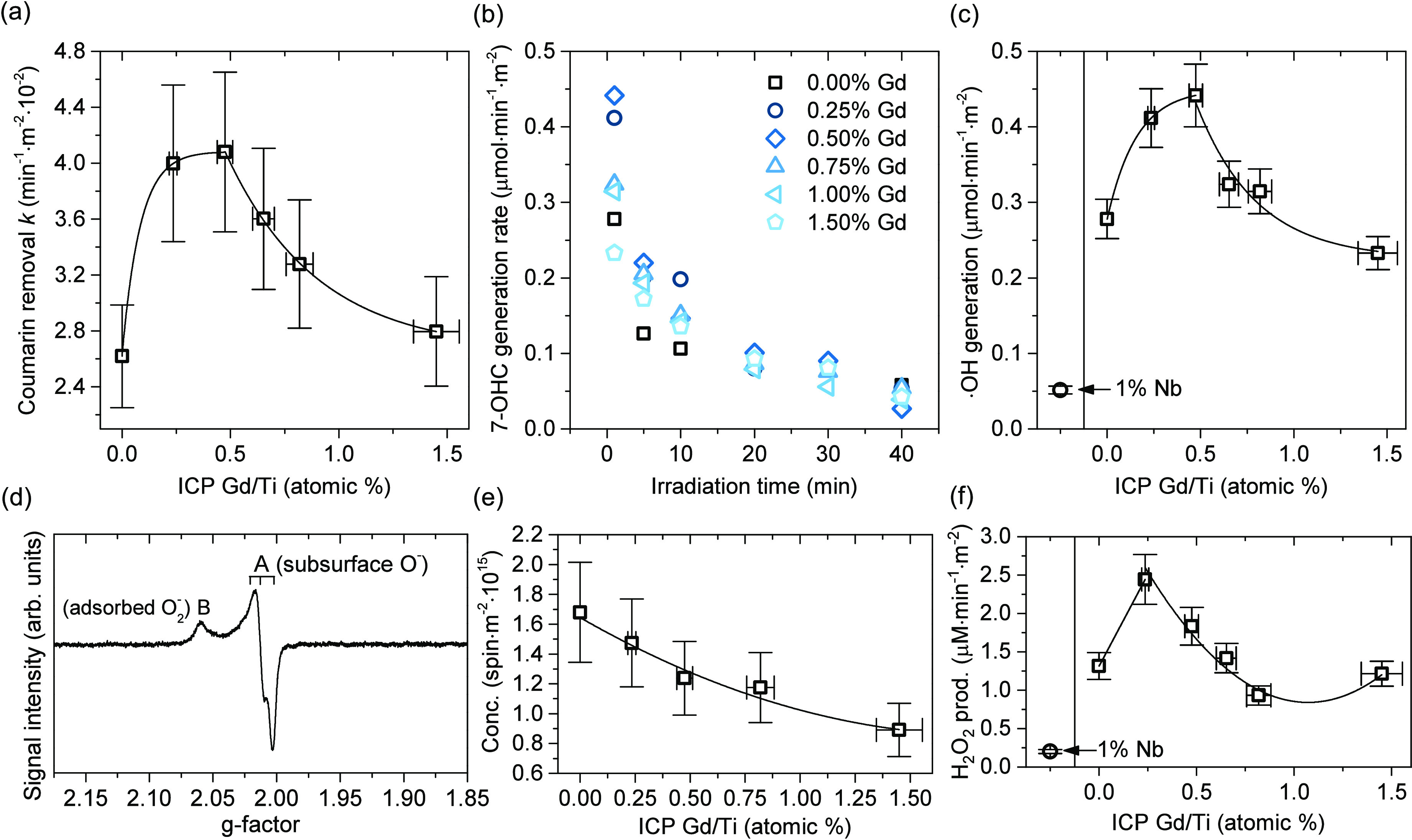
Surface-normalized results of photocatalytic coumarin degradation (a) as well as the corresponding rate of 7-hydroxycoumarin (7-OHC) accumulation (b) and ·OH generation rate, estimated from the initial 7-OHC formation (c). EPR spectrum of the stable h^+^ trapped radicals, generated after in situ UV-light irradiation for all samples (d), together with the estimated concentration of the trapped holes after considering the surface area of the sample (e); see text for details. Determined H_2_O_2_ production rate from the H_2_O/O_2_ system with isopropanol acting as a hole scavenger (f).

Based on the obtained results, doping of the TiO_2_ octahedra with Gd has two effects. First of all, it reduces the number of free electrons, markedly decreasing their ability to reduce 4-nitrophenol when compared to the unmodified structure. However, simultaneously, it visibly increases the degradation rate of phenol, when introduced in a low amount. Therefore, as the final part of this study, the Gd effect on the ability to generate reactive species by the prepared samples was further investigated. This was started with additional degradation tests of coumarin and simultaneous monitoring of the emission associated with the generation of 7-hydroxycoumarin (7-OHC), which is a known probe for the generation of the ·OH radicals by the TiO_2_ photocatalysts.^63,64^ Obtained results are shown in Figure 8, with detailed data presented in Figure S12 in the Supporting Information. As observed, the degradation rate of the coumarin removal during the 40 min process follows the same trend as phenol, additionally confirming overall results. Moreover, this is strictly connected with the initial (1 min) rate of 7-OHC generation, as presented in Figure 8b. In the case of 7-OHC generation, analysis of the initial rate is preferred, since in the later parts of the process, simultaneous degradation of remaining coumarin, created 7-OHC, and other byproducts, makes it difficult to meaningfully compare obtained results. This is shown well when comparing the 7-OHC generation rates for different reaction times, which shows that the highest differences are observed at the start of the process. Nevertheless, the positive effect of the Gd introduction on the ·OH generation rate is easily observed up to the 10 min of the process, which corresponds well with the results observed both for phenol and coumarin itself. The final estimated ·OH generation rate is presented in Figure 8c. Finally, additional experiments for the 1% Nb-doped sample clearly showed that a further increase of the free electrons follows the trend and significantly reduces ·OH generation, in accordance with the previous findings on negligible Nb^5+^ effect on the phenol degradation ability by similar structures.

In this regard, it can be noticed that the compromised reduction ability of the Gd-doped {1 0 1} facets is beneficial for ·OH generation by these structures. This fact can be intuitively connected with the water oxidation that produces ·OH through the involvement of h^+^. Therefore, we further investigated the fate of photogenerated holes by measuring EPR spectra under *in situ* UV light illumination. As presented in Figure 8d, after subtraction of the initial spectrum, a stable signal of the h^+^ trapped species was clearly observed at *g* = 1.975–2.075. Analogical signals were observed previously for the TiO_2_ samples by multiple authors^65−68^ and can be attributed to the subsurface lattice O^–^ species (signal A with *g*_1_ ≈ 2.003, *g*_2_ ≈ 2.013, and *g*_3_ ≈ 2.019) and adsorbed O_2_^–^ (species B, *g* ≈ 2.059, with other components commonly not observed). Analogical spectra were observed for all investigated samples, without qualitative differences; however, their relative intensity was clearly reduced together with the Gd concentration. Noteworthily, as presented in Figure 8e, after considering the available surface area of each material, the estimated concentration of these species forms a clear trend with respect to Gd presence, which is in strict correlation with the determined donor densities. This phenomenon is readily explained based on the existing information. First of all, as shown by Shirai et al., hole trapping on anatase surfaces is a complex process and the stability of the specific trapping site depends heavily on the local electron density.^69^ Noteworthily, in the case of the {1 0 1} facets, the suitable h^+^ trapping is often reported to occur at the three-coordinated O^2–^ atoms near the surface.^69−71^

This agrees with the observed species A, which was the dominant signal generated for all prepared samples. However, despite its subsurface nature, this signal is still sensitive to the surface presence of adsorbed species, which further stabilize trapped h^+^.^66,72^ This connects especially with the surface presence of water and hydroxyl groups, which are known to heavily influence on the hole trapping and resulting photocatalytic activity^73,74^ (especially the −OH presence due to their higher electron density^69^). In this regard, systematic reduction of the A intensity suggests that the surface became less hydrophilic with the increased Gd amount, reducing the number of hydroxyl groups and in return destabilizing the trapped h^+^ state. Noteworthily, this interpretation not only explains the nature of the EPR signal change but also connects it strictly with the concentration of excess electrons, determined from Mott–Schottky analysis. This is due to the low energy of the perfect (1 0 1) surfaces, which is known to reduce spontaneous H_2_O dissociation and resulting hydroxylation of these facets.^75^ On the other hand, Setvin et al. have observed the formation of basic hydroxyls in the STM images of the (1 0 1) surface as the result of multiple-electron oxygen reduction, which could be summarized with possible surface reactions (1–3):^76^
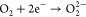
1
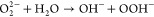
2
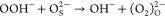
3where O_s_^2–^ is oxygen in the bridging surface lattice site and (O_2_)_O_^2–^ is an oxygen molecule replacing O_s_^2–^. As shown through the DFT calculations, the occurrence of these reactions is strictly dependent on the presence of excess electrons and the local surface configuration. Specifically, OOH^–^ dissociation (3) was shown to be the limiting step of the overall process, with a significant stabilizing role of the neighboring −OH groups due to their higher affinity to electrons.^76^ Interestingly, the same study highlights OOH^–^ as a possible substrate to H_2_O_2_, in accordance with reaction 4:
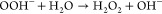
4Although it must be noted that other studies report slightly different paths to H_2_O_2_ formation, they all involve generation and further transformation of analogical peroxo species, e.g., according to reactions (5–7), involving two subsequent H^+^ transfers:^77,78^
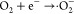
5
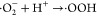
6
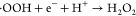
7

Generation of H_2_O_2_ is known to be an important process during photodegradation of organic pollutants, where it can especially form hydroxyl radicals due to the homolysis of the O–O bond (8) or dissociate to the −OH upon further reduction (9 and 10):
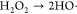
8
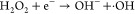
9
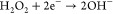
10

In this regard, hydroxylation of the {1 0 1} surfaces is strictly connected to the oxygen reduction. Furthermore, formation of both H_2_O_2_ and possible peroxo species ·OOH/OOH^–^ is a centerpiece of the different transformation pathways. Especially, possible dissociation of OOH to form surface −OH (2) or their alternative reaction with H_2_O/H^+^ to form H_2_O_2_ (4 and 7) might be seen as a main competing process between all possibilities.^79^ Therefore, we have performed additional tests of H_2_O_2_ generation over the prepared samples under slightly modified conditions (increased aeration and addition of 2-propanol) in order to get better insight into this competition. As presented in Figure 8f, the analogical maximum of the generation rate was observed also in this case; however, it was clearly shifted to the 0.25% doped sample, instead of the 0.50% one. This is observed independently on the ·OH generation. Therefore, H_2_O_2_ dissociation (8 and 9) is not a predominant source of the ·OH. This is specifically suggested due to both increased amount of the H_2_O_2_ and higher density of donor states observed for the 0.25% sample, compared to 0.50%, which should promote occurrence of both reactions 8 and 9. However, these features were not supported by the highest coumarin hydroxylation, disproving decisiveness of such mechanism. Ultimately, based on the summarized results, the probable source of the increased degradation ability upon Gd doping is suggested as follows:

1. For the unmodified sample, relative abundance of the free electrons results in a higher surface hydroxylation, compared to the Gd-doped materials, which stabilize h^+^ trapping as the subsurface O^–^. Simultaneously, possible dissociation of OOH^–^ (or analogical peroxo species) is partially hindered by the −OH presence. Samples show relatively high photocatalytic activity.
2. Starting from this point, introduction of Gd systematically decreases the density of free electrons, which reduces surface hydroxylation and h^+^ trapping. For the lowest Gd concentration, this promotes H_2_O_2_ formation, possibly both due to the increased presence of nondissociated H_2_O at the surface, facilitating reaction (4), and the decreased amount of surface −OH promoting electron reaction with possible ·OOH (7).
3. Further increase of the Gd concentration decreases H_2_O_2_ generation but maximizes ·OH production. This results from further destabilization of the OOH^–^ upon reduction of the surface −OH amount, which shifts the mechanism from H_2_O_2_ generation to peroxo dissociation, analogical to 3. Simultaneously, photogenerated holes effectively oxidize remaining hydroxyls to generate ·OH with the optimal rate, providing efficient phenol degradation. Noteworthily, alternative explanation of the increased ·OH generation as the result of efficient H_2_O_2_ consumption is unlikely, since it should result either from its photolysis (8) or from its further reduction (9) and, these cannot explain the lower ·OH generation for the 0.25% sample, as discussed above.
4. For even higher Gd amounts, overall activity starts to decrease as the result of both progressed dehydroxylation, hindering −OH oxidation to ·OH, and reduced number of excess electrons available to promote reactions 1–9.

**Figure 9:**
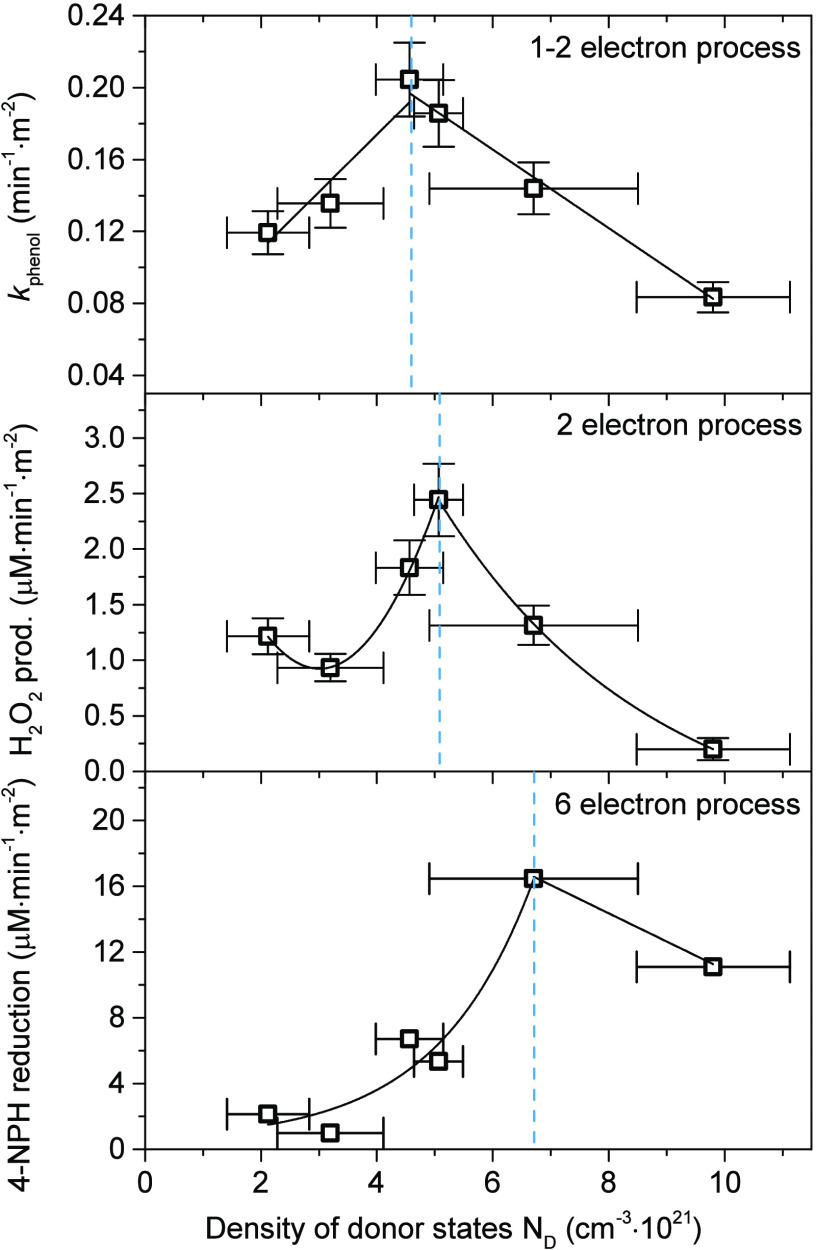
Relation between the observed, surface-normalized rate constant of phenol degradation, H_2_O_2_ generation, and 4-nitrophenol reduction vs calculated density of donor states for overall series of the {1 0 1} exposing TiO_2_ nanoparticles.

Such a mechanism explains well the importance of cooperation between electrons and holes on the {1 0 1} atom during the water treatment process. However, it simultaneously implies that the highest efficiency should be obtained when both charge carriers react efficiently to promote a specific pathway of oxygen reduction/water oxidation. This inseparably connects with the number of exchanged electrons needed to conduct the desired reaction, which in the case of the H_2_O/O_2_ systems presents an exceptionally complex picture of the possible surface interactions and reaction pathways. In this regard, the presented results show an easy approach to optimize this problem through controlled *N*_D_ manipulation with suitable dopants. As shown in Figure 9, the observed reaction efficiencies formed clear maximum concerning *N*_D_ for all tested reactions, simultaneously creating a trend with respect to the number of electrons needed to conduct desired process.

## 4 Conclusions

In the present study, Gd-doped anatase nanoparticles, enclosed with the {1 0 1} facets, were prepared via two-step synthesis from the P25 TiO_2_ and K_2_Ti_6_O_13_ intermediate phase. The Gd incorporated into the TiO_2_ lattice acts as an electron-accepting dopant, as evidenced by the DFT calculations; XRD, TEM, and XPS measurements; absorption spectra; and Mott–Schottky analysis of the samples. The electron-accepting character of Gd leads to the systematic lowering of the density of donor states (excess e^–^), resulting in surface hydroxylation and stability of the h^+^ trapping at the subsurface O^2–^ sites. This allowed us to fine-tune photocatalytic activity of the {1 0 1} facets in a series of specific processes such as phenol degradation, ·OH generation (coumarin based), O_2_ reduction to H_2_O_2_, and 4-nitrophenol reduction to 4-aminophenol. The clearly observed maxima of all tested reactions confirm the importance of both charge carriers on the final photocatalytic activity, simultaneously showing that a specific pathway might be promoted as the result of optimized ground-state density of excess charge. Specifically, reactions that require more electrons to occur achieved maximum rates over materials that show a higher concentration of donor states. Besides achieving high activity in case of phenol degradation, exceeding P25 standard by over 52% with observed photonic efficiency of 2.19% under Xe lamp irradiation, which due to our best knowledge is one of the fastest degradation rates reported so far for a purely photocatalytic system, the presented results suggest that such a facet-dopant optimization of a photocatalyst might be promising for other materials and applications as well.

## Supporting Information Available

Detailed analysis of the morphology; results of the Rietveld refinement; repeated synthesis of the selected samples and error estimation detailed XPS results; detailed results of X-ray photoelectron spectroscopy measurements; additional TEM images; additional UV–vis spectra, PL spectra, and detailed Mott–Schottky graphs; detailed kinetic data for phenol degradation, coumarin hydroxylation, and 4-nitrphnoel reduction; and data for the control Nb-doped sample (PDF)
