# Supplementary material for: Fine-Tuning the Photocatalytic Activity of the Anatase {1 0 1} Facet through Dopant-Controlled Reduction of the Spontaneously Present Donor State Density
Source: ACS Mater Au. 2024 Mar 26;4(4):436–49. doi: 10.1021/acsmaterialsau.4c00008 (PMC11240422; doi:10.1021/acsmaterialsau.4c00008)
Supplement: Supplementary file 1 — mg4c00008_si_001.pdf [file mg4c00008_si_001.pdf]

Supporting Information:
Fine-Tuning Photocatalytic Activity of the Anatase {1 0 1} Facets Through the Dopant-Controlled Reduction of Spontaneously Present Donor States Density

Szymon Dudziak,*[a] Jakub Karczewski,[b] Adam Ostrowski,[c] Grzegorz Trykowski,[d] Kostiantyn Nikiforow[e] and Anna Zielińska-Jurek[a]

[a.] *Department of Process Engineering and Chemical Technology, Gdansk University of Technology, Narutowicza 11/12, Gdańsk 80-233, Poland.*
[b.] *Institute of Materials Science and Nanotechnology, Gdansk University of Technology, Narutowicza Street 11/12, Gdańsk 80-233, Poland.*
[c.] *Institute of Molecular Physics, Polish Academy of Sciences, Smoluchowskiego 17, Poznań 60-179, Poland.*
[d.] *Department of Chemistry of Materials, Adsorption and Catalysis, Faculty of Chemistry, Nicolaus Copernicus University, Gagarina 7, 87 100 Torun, Poland.*
[e.] *Institute of Physical Chemistry, Polish Academy of Sciences, Kasprzaka 44/52, 01-224, Warsaw, Poland.*
* Correspondence: szymon.dudziak@pg.edu.pl

1. Analysis of the octahedrons morphology

Observed morphology of the prepared TiO_2 octahedrons was compared to the theoretical crystal shape of the particle enclosed with the family of {1 0 1} planes. Visualisation of the crystal was performed using VESTA software [1], based on the available .cif file of the anatase structure [2]. **Figure S1** show the theoretical orientation between exposed facets (a), as well as observed ones for all samples (b). Please note that for the real samples, some deviation between observed and theoretical angles is expected due to the crystal's orientation in space.

a)

b)

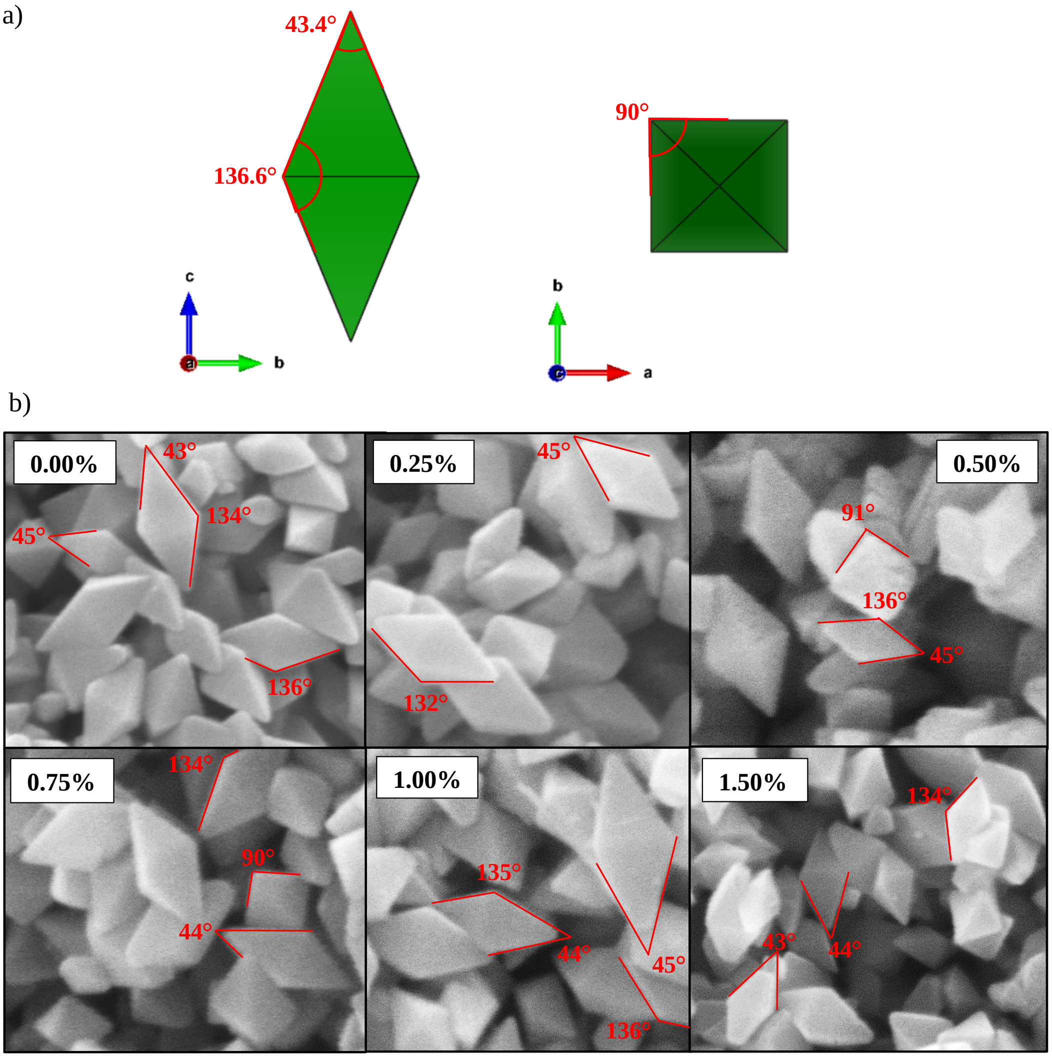

Figure S1. Comparison between theoretical (a) and observed crystal shapes of anatase octahedrons (b).

2. Rietveld refinement

Figure S2 shows results of Rietveld refinement of the XRD patterns measured for the unmodified and Gd-doped samples.

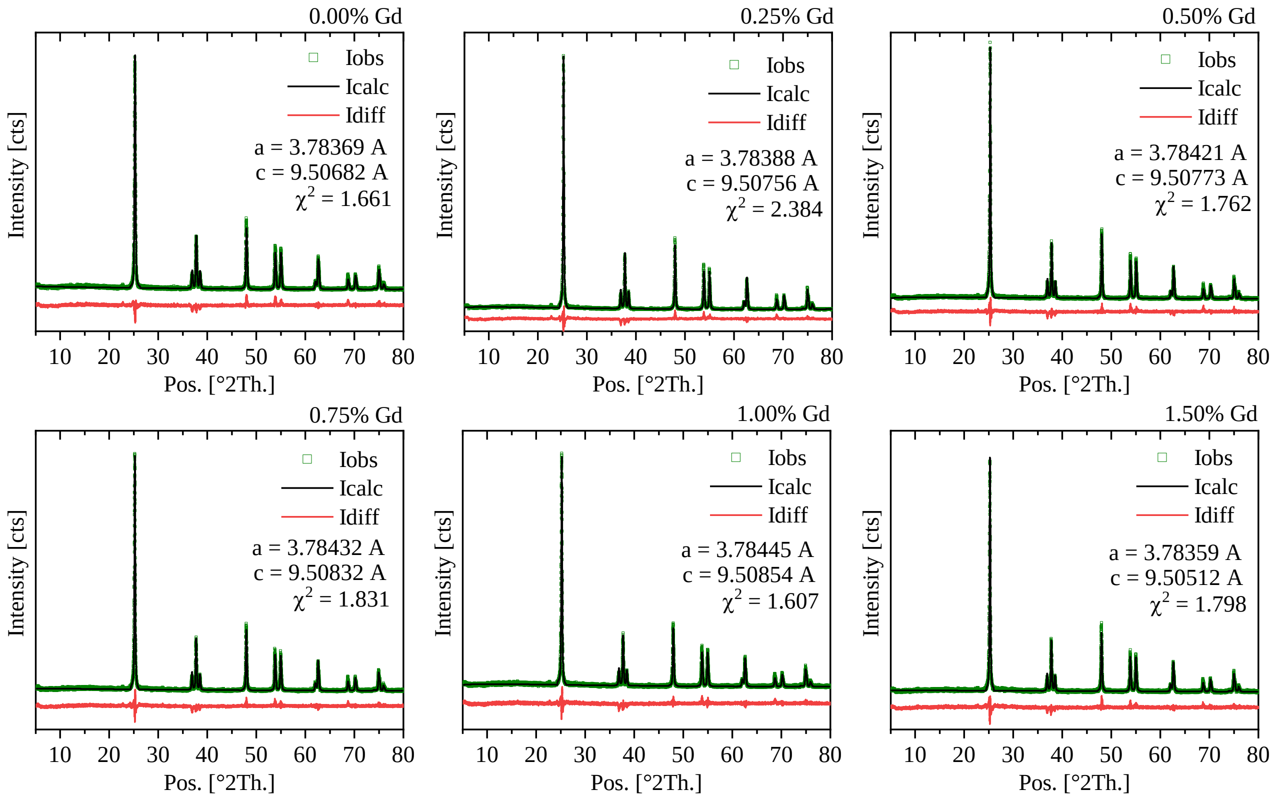

Figure S2. Results of the Rietveld refinement of the obtained XRD patterns for the Gd-doped samples.

3. Resynthesis of the 0.75% sample and error estimation for phenol degradation

For a better error estimation during the phenol degradation, a sample with a nominal 0.75% Gd amount was resynthesized to account for the random error associated with the preparation procedure. The resynthesized sample was checked using XRD and SEM/EDS analyses, which are shown in **Figure S3**. Analogical resynthesis was performed also for a 0.00% sample for better error estimation during phenol degradation. Analogically to sample 0.75% Gd, the XRD pattern did not show any significant changes. Detailed SEM and EDS studies were not performed in this case.

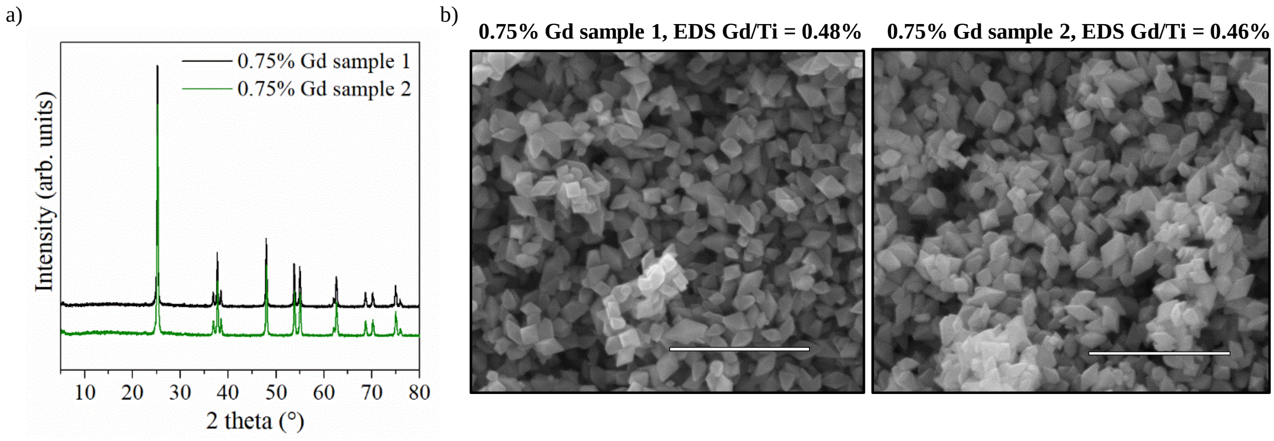

Figure S3. Comparison of the XRD patterns and SEM/EDS results of the two 0.75% samples, prepared for the error estimation. All observed reflections correspond to the anatase TiO_2. Scale bars are 1 µm.

Estimation of the error associated with the overall procedure of phenol degradation was made based on the repetitions made in the same process conditions for the unmodified sample, sample with nominal Gd/Ti = 0.75% (in the middle of the investigated range) and commercial P25. In the case of the self-prepared samples, repetitions included the resynthesis of the material, as highlighted above. **Table S1** show the summation of these tests.

Table S1. Error estimation during the photocatalytic degradation of phenol.

Sample	Repetition	BET ($m^2 \cdot g^{-1}$)	Observed *k* (min^{-1})	Normalised *k* ($min^{-1} \cdot m^{-2}$)	Mean ($min^{-1} \cdot m^{-2}$)	Δ*k* ($min^{-1} \cdot m^{-2}$)	Relative error (%)
0.00%	1	17.2	0.126	0.147	0.144	0.006	4.2
	2	18.9	0.133	0.141			
0.75%	1	15.4	0.123	0.160	0.153	0.015	9.8
	2	14.2	0.103	0.145			
P25	1	52.7[a]	0.107	0.041	0.042	0.002	4.8
	2		0.115	0.043			

[a]Mean from two measurements.

Based on the presented results, possible error during the photocatalytic degradation of phenol was estimated in a relative form (the ratio between the Δ*k* and its mean value). The final relative error goes up to 10%, which is a value adapted during the study for all samples. Analogical error estimation for surface-normalised TOC removal was based on the repeated synthesis of the 0.75% sample and two measurements of the P25 standard. Obtained relative errors, calculated in a way analogical to *k*, gave values of 14% for the 0.75% sample and 6% for P25. From these values, the higher one was adapted for the overall study.

In the case of coumarin degradation and 7-hydroxycoumarin generation, error estimation was based only on the two measurements of the 0.75% samples. Analogical error values were estimated as 13.9% and 9.4%, respectively.

For other measurements, the doubled samples 0.00% and 0.75% were mixed and used as one material.

4. Detailed results of X-ray photoelectron spectroscopy measurements

Figure S4 shows detailed XPS results of the observed Ti, O and C states for the prepared samples, while additional numerical data presented in **Table S2**.

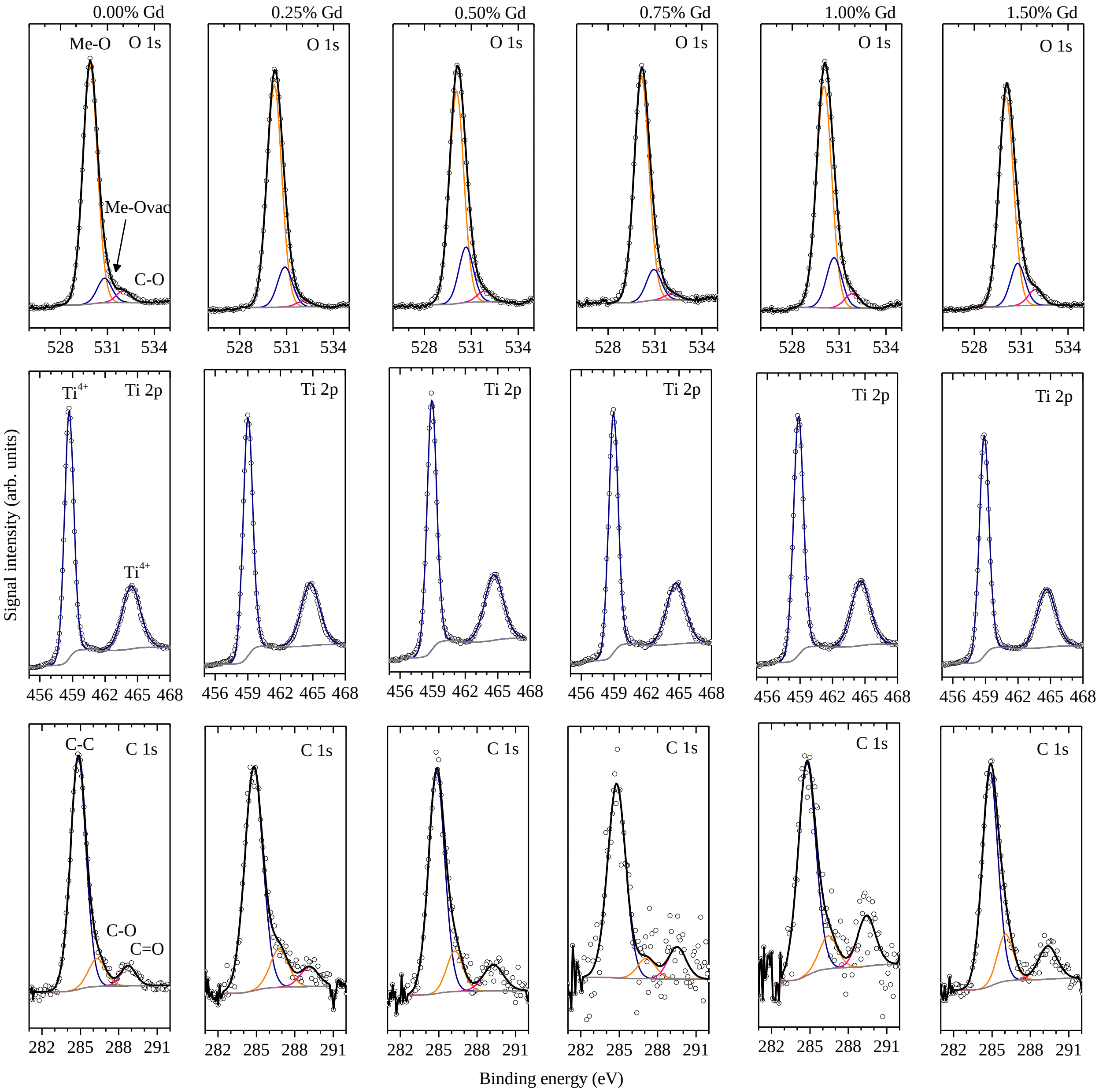

Figure S4. XPS signal of O 1s, Ti 2p and C 1s states for the prepared samples.

Table S2. Numerical XPS data for the prepared samples.

Sample	Ti, %at	O (Me-O), %at	O (Me-Ovac), %at	Gd, %at	C, %at	Ti2p3/2, eV	Gd 4d, eV	Gd 3d, eV	O1s (Me-O), eV
0.00% Gd	23.49	50.97	5.21	0	17.85	458.69	-	-	529.9
0.25% Gd	26.29	53.52	9.67	0.46	8.63	459.02	143	1187.9	530.22
0.50% Gd	24.81	46.45	12.15	1.18	13.07	458.92	142.8	1187,6	530.06
0.75% Gd	27.07	56.61	7.76	1.2	5.78	458.95	142.9	1187.7	530.15
1.00% Gd	26.81	51.05	11.61	1.79	5.41	458.86	142.8	1187.7	530.04
1.50% Gd	24.12	49.01	9.92	2.15	11.07	458.88	142.7	1187.7	530.05

5. Additional transmission electron microscope images

Figure S5 shows additional TEM images of the analyzed 0.00%, 0.25% and 0.50% samples.

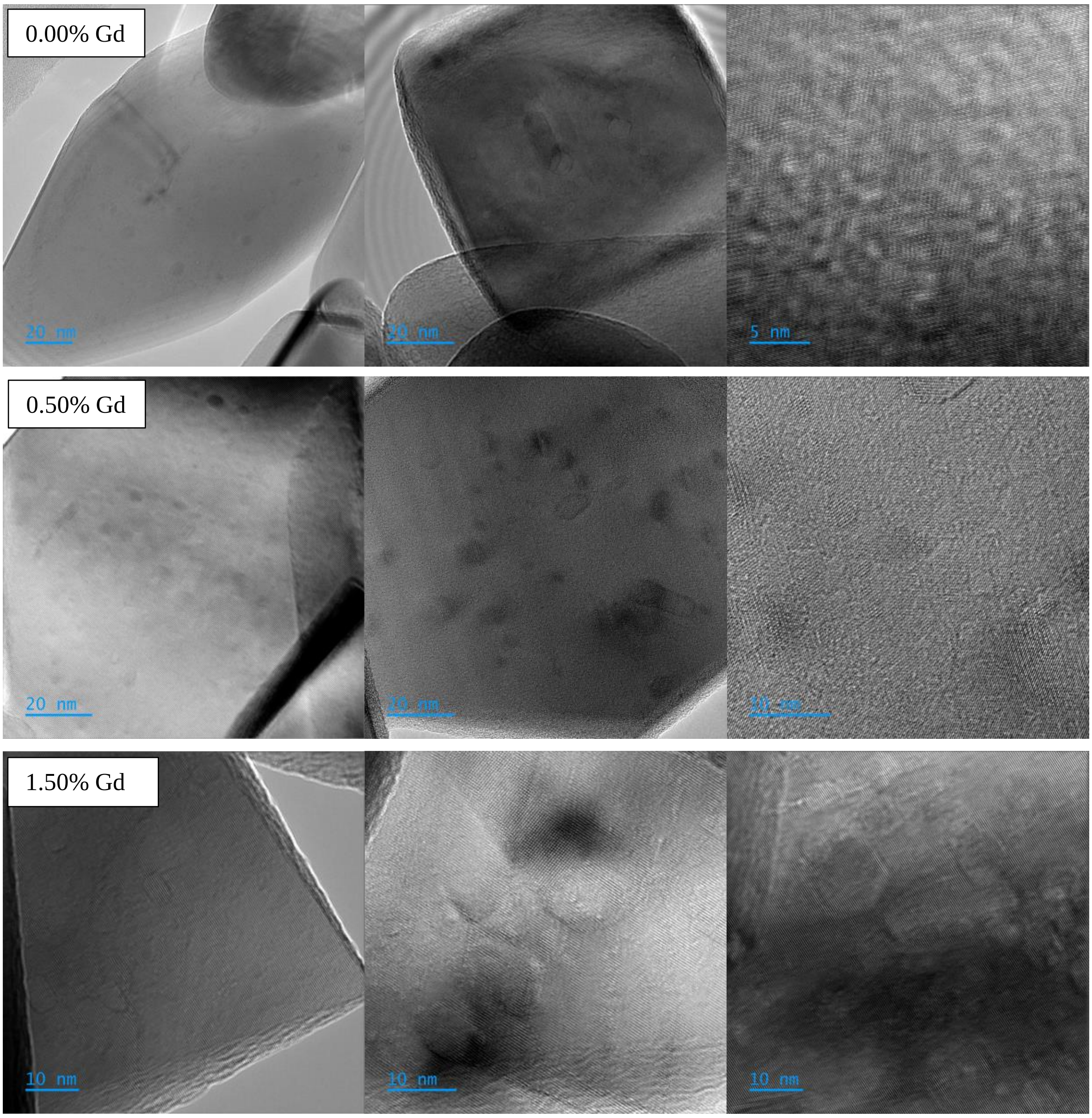

Figure S5. Additional TEM images of the selected samples.

6. Detailed results of phenol degradation and 4-nitrophenol reduction

Figure S6 shows detailed results of the photocatalytic degradation of phenol for all of the {1 0 1} samples, together with the observed formation of its aromatic by-products. No benzoquinone presence was observed for all samples. Furthermore, **Figure S7a** show the subsequent transformation of the measured phenol concentration to obtain the *k* value from the ln(C_0/C) plot, as well as the observed reduction of 4-nitrophenol for the prepared samples. Data for 0.00% (phenol) and 0.75% (phenol and 4-nitrophenol) samples are shown as a mean from two measurements.

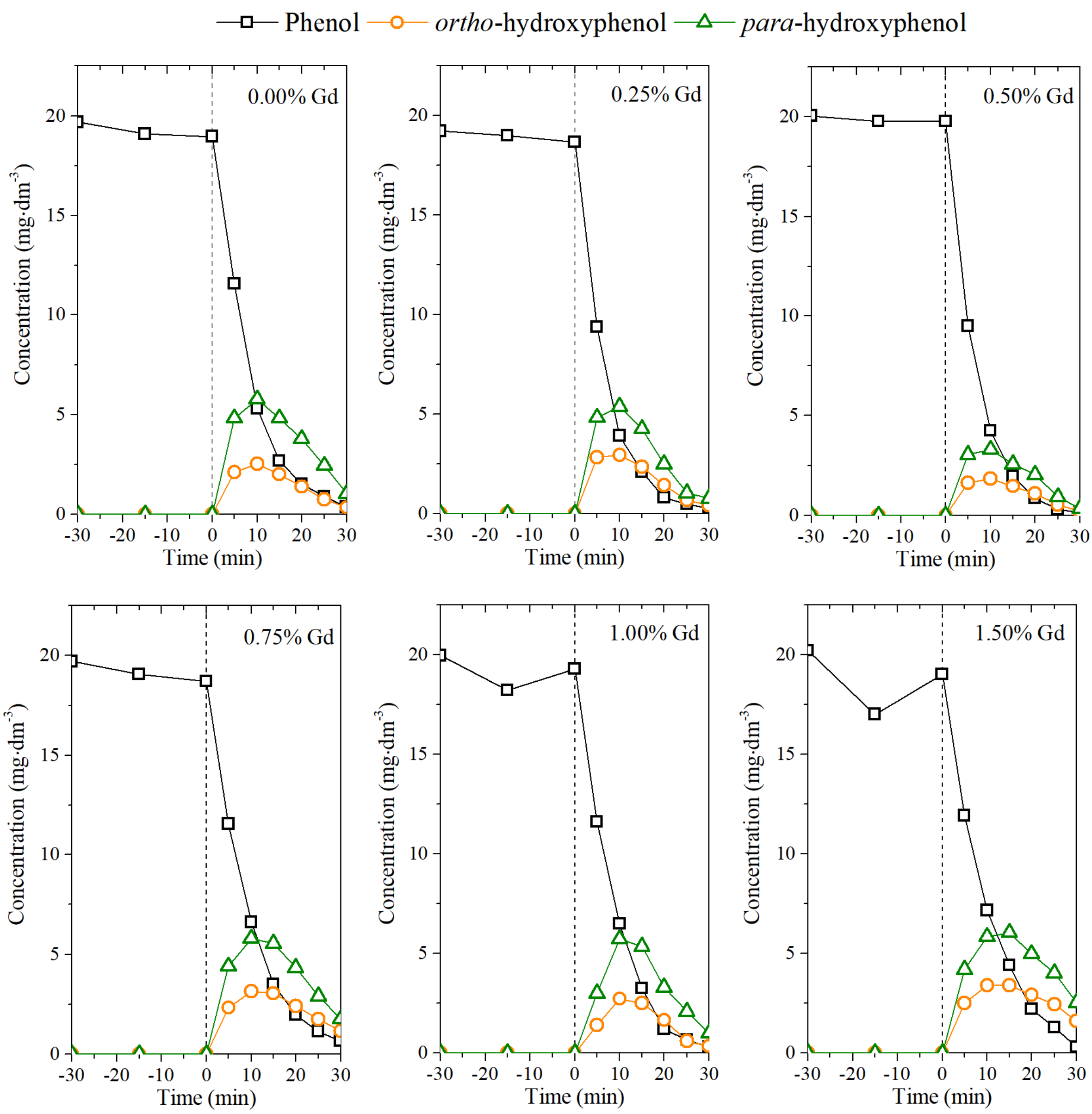

Figure S6. Detailed results of the photocatalytic degradation of phenol, for the {1 0 1} enclosed samples.

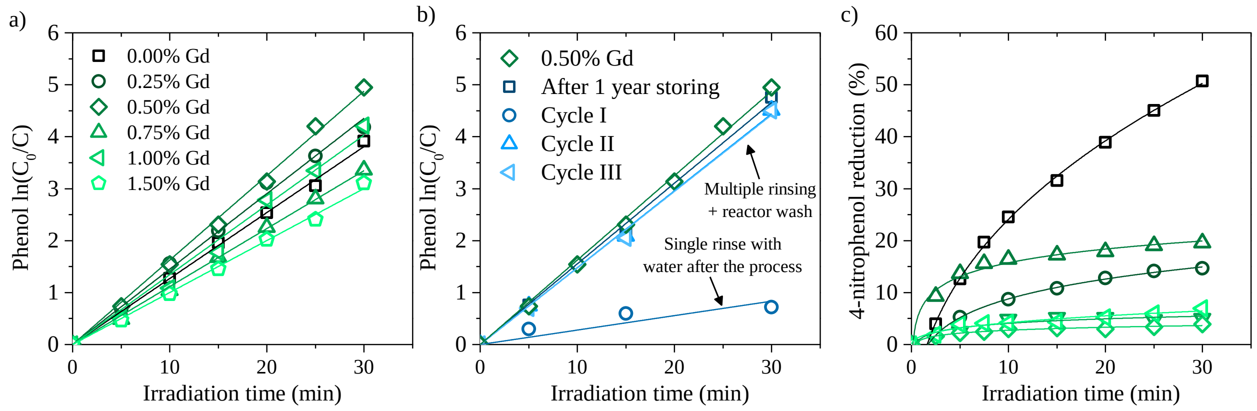

Figure S7. Phenol ln(C_0/C) plot, with linear fitting to obtain k value for the I-order reaction (a), as well as an observed 4-nitrophenol reduction for the {1 0 1} enclosed samples (c).

8. UV-Vis absorbance spectra

The individual absorbance spectra for each sample, as obtained, are shown in **Figure S8** below.

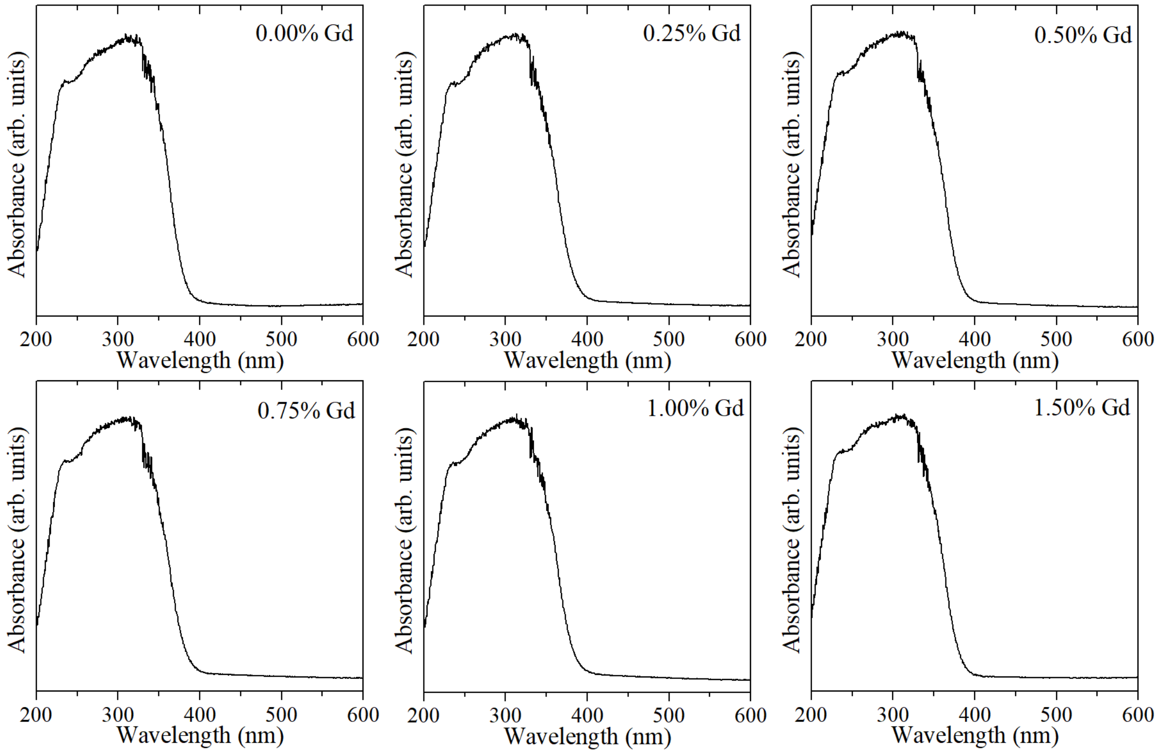

Figure S8. Individual UV-Vis absorbance spectra for each sample.

9. Control sample doped with 1% of Nb^{5+}

An additional sample, doped with a nominal 1% of Nb^{5+}, was prepared as the control to investigate the $k(N_D)$ relation within the higher N_D range. The preparation procedure, based on the previous study [3], was strictly analogical to the one described in the present work, with the corresponding amount of $NbCl_5$ introduced in place of gadolinium nitrate. Control measurements included XRD, UV-Vis spectra, SEM, BET surface area, donor density from the Mott-Schottky plot, phenol degradation and ·OH generation. A summation of these results is shown in **Figure S9**.

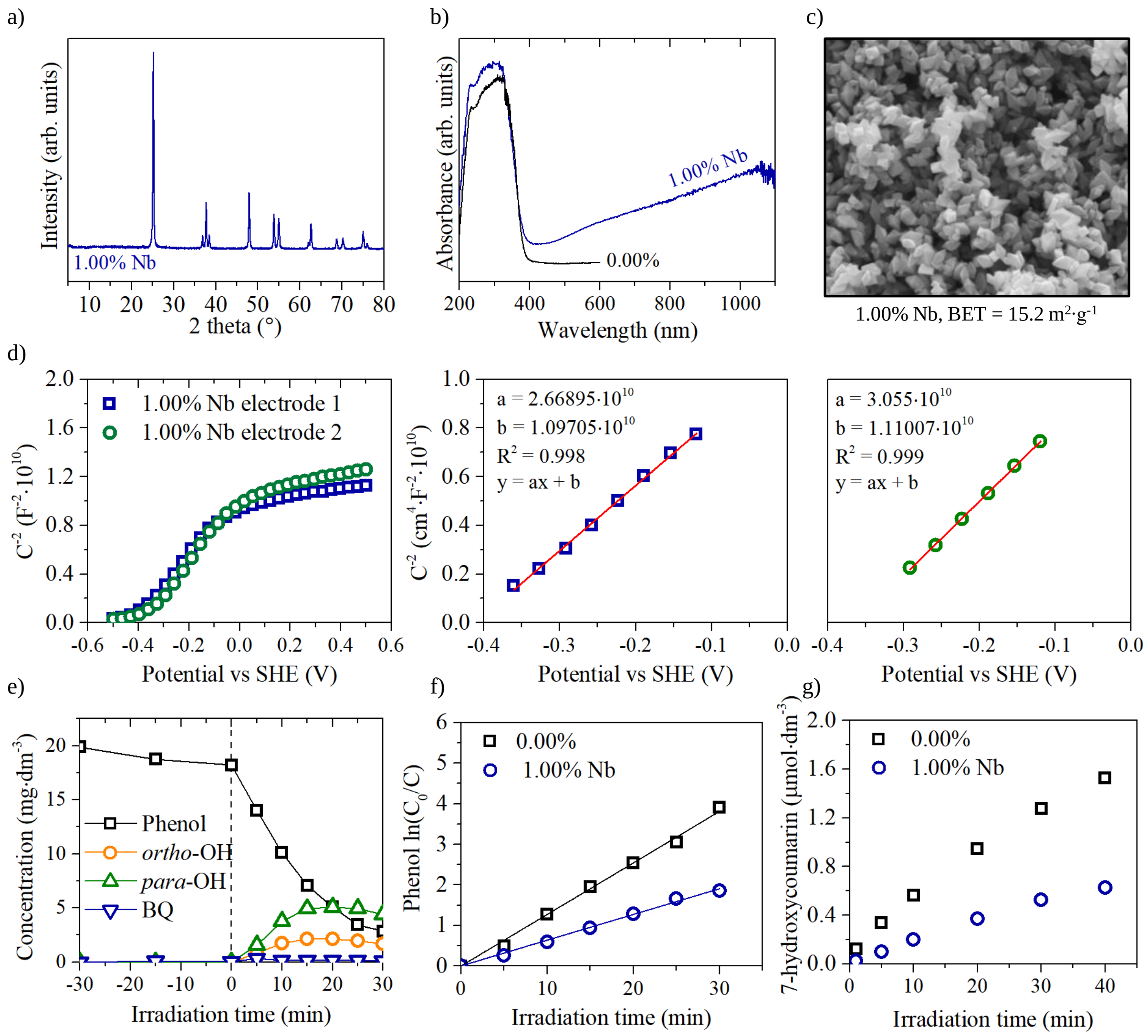

Figure S9. Detailed results of the control measurements, performed for the sample doped with 1% at. of Nb. XRD pattern (a); UV-vis absorbance spectrum, compared to the unmodified sample (b); SEM image and corresponding BET surface area (c); results of Mott-Schottky analysis and corresponding fitting of the slope connected with donor density (d); phenol degradation results, where BQ stands for benzoquinone (e); rate constant fitting of the phenol degradation, compared to the unmodified sample (f); and observed results of the 7-hydroxycoumarin formation, compared to unmodified sample (g).

10. Mott-Schottky analysis

Detailed results of the Mott-Schottky analysis, obtained for every separate electrode, are presented in **Figure S10**. Panels (a, c, e, g and i) show overall plots and panels (b, d, f, h and j) show the results of performed fitting.

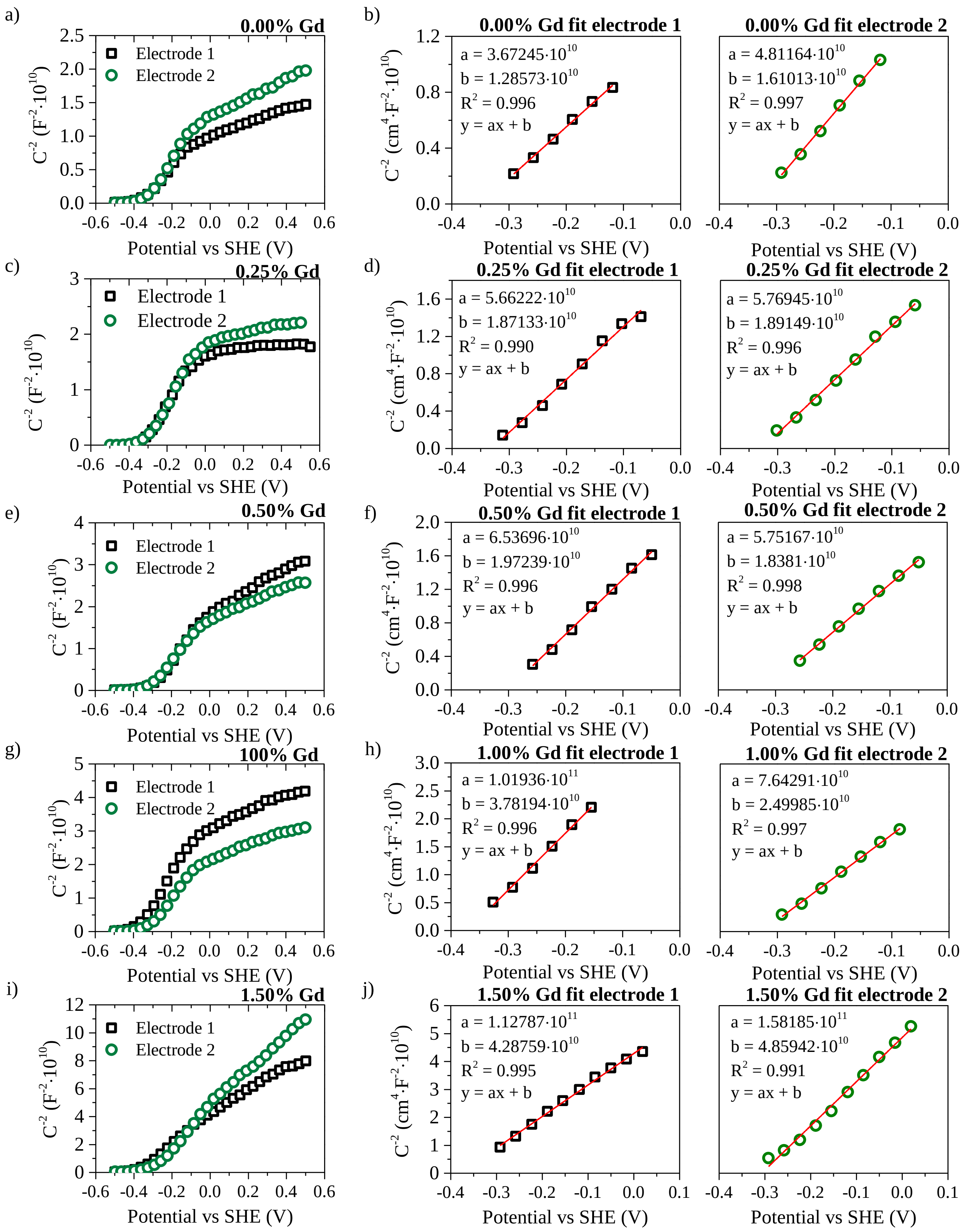

Figure S10. Detailed results of the performed Mott-Schottky analysis, including overall plots (a, c, e, g) and results of the performed fitting (b, d, f, h).

11. Photoluminescence spectra (PL)

Recorded photoluminescence spectra of the prepared materials are shown below in **Figure S11**. No significant and/or systematic reduction of the emission is observed as the result of Gd introduction, suggesting that charge transfer from TiO_2 to possible Gd species is not the main mechanism of the observed changes in photocatalytic activity.

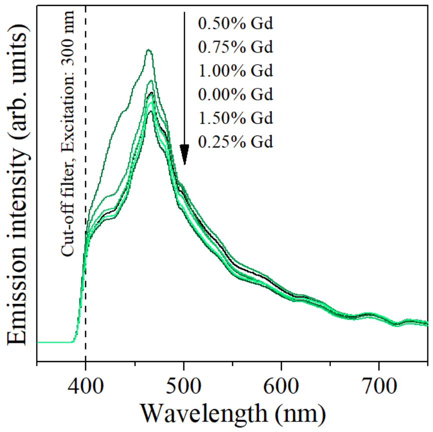

Figure S11. Photoluminescence spectra of the prepared TiO_2 materials, modified with Gd. Wavelengths shorter than 400 nm were cut off during the measurement. Excitation with 300 nm wavelength.

12. Detailed results of coumarin degradation and 7-hydroxycoumarin formation

Detailed results of the performed coumarin degradation and 7-hydroxycoumarin formation are shown in **Figure S12**. Rate constants of coumarin degradation were calculated assuming I-order reaction kinetics, analogically to phenol.

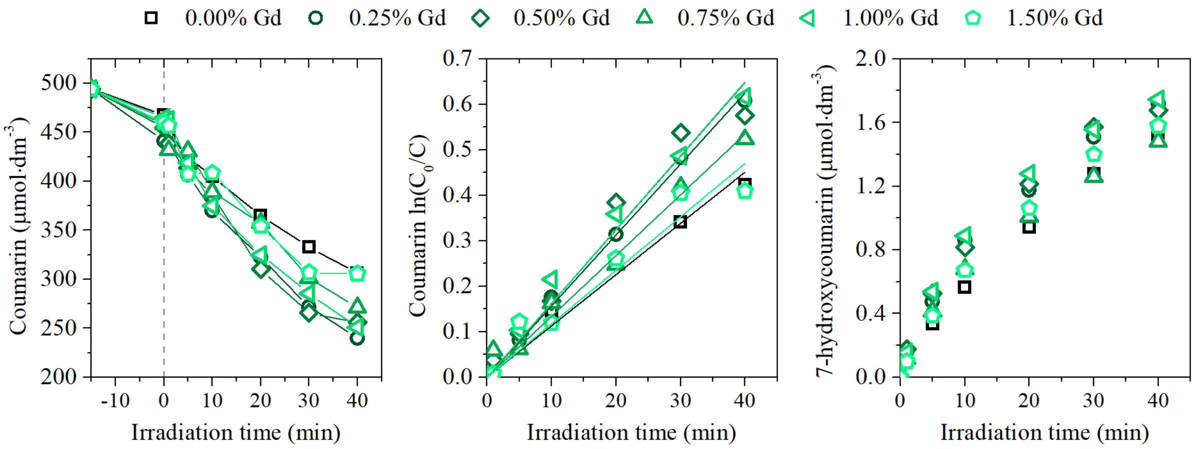

Figure S12. Detailed results of the coumarin degradation and 7-hydroxycoumarin formation.

13. Additional references for Supporting Information

1 K. Momma and F. Izumi, *J Appl Crystallogr*, 2011, **44**, 1272–1276.

2 C. J. Howard, T. M. Sabine and F. Dickson, *Acta Crystallogr B*, 1991, **47**, 462–468.

3 S. Dudziak, E. Kowalska, K. Wang, J. Karczewski, M. Sawczak, B. Ohtani and A. Zielińska-Jurek, *Appl Catal B*, , DOI:10.1016/j.apcatb.2023.122448.
